# Identification of small molecule inhibitors for the *Brachyspira pilosicoli* glutamate racemase (Bp-MurI) enzyme using a computational and experimental approach

**DOI:** 10.1038/s41598-026-46506-w

**Published:** 2026-04-02

**Authors:** Ravi Kant, Roberto La Ragione, Myron Christodoulides

**Affiliations:** 1 Molecular Microbiology, School of Clinical and Experimental Sciences, Faculty of Medicine, University of Southampton, Southampton, SO16 6YD England; 2 Faculty of Applied Sciences & Biotechnology, Shoolini University, Solan, 173229 Himachal Pradesh India; 3 School of Biosciences, Faculty of Health and Medical Sciences, University of Surrey, Edward Jenner Building, Guildford, GU2 7XH England; 4 School of Veterinary Medicine, Faculty of Health and Medical Sciences, University of Surrey, Guildford, GU2 7AL England

**Keywords:** *Brachyspira pilosicoli*, intestinal spirochaetosis, glutamate racemase, computational virtual screening, molecular docking, molecular dynamics simulations, small molecule inhibitors, Maybridge, Biochemistry, Computational biology and bioinformatics, Drug discovery

## Abstract

*Brachyspira pilosicoli* (Bp) causes intestinal spirochaetosis in poultry and pigs and more rarely in humans. In this pilot study, we used a computational approach to identify potential small molecule inhibitors for the Bp glutamate racemase (Bp-MurI), an essential enzyme for cell wall biosynthesis, and then tested the Hit compounds against *B. pilosicoli* in a broth microdilution assay. Protein modelling and validation generated a reliable three-dimensional structure for Bp-MurI and development of a structure-based pharmacophore model led to the virtual screening of > 51,000 drug-like compounds from the Maybridge High Throughput Library. After filtering and molecular docking, three top Hit compounds (Hit 1, HTS02114; Hit 3, AW00718; Hit 4, HTS04172) were selected based on their binding affinities and interactions with the active site residues. Molecular Dynamics Simulations assessed the stability and dynamics of the Bp-MurI-inhibitor complexes. Hit 4 showed no biocidal activity against *B. pilosicoli* (MIC>1mM), Hit 1 had weak activity (MIC50 of 0.5 mM), and Hit 3 was the most effective (MIC50 and MIC100 values of 0.25 and 0.5 mM, respectively). Tiamulin, the currently used antibiotic, was highly effective (MIC50 and MIC100 values of 0.015 µM and 2µM, respectively). None of the compounds were cytotoxic to an epithelial cell line in culture using a resazurin assay, and SwissADME favourably assessed their physicochemical, pharmacokinetic and drug-likeness properties in silico. In conclusion, a computational modelling approach coupled with high throughput screening in silico is a useful process for more in-depth drug discovery against this pathogen.

**Supplementary Information:**

The online version contains supplementary material available at 10.1038/s41598-026-46506-w.

## Introduction

*Brachyspira pilosicoli* is a zoonotic pathogen of global distribution that causes avian intestinal spirochaetosis (AIS)^1^, porcine intestinal spirochaetosis (PIS)^2^ and more rarely human intestinal spirochaetosis (HIS)^3^. *B. pilosicoli* (Bp) is a slow-growing, flagellated, anaerobic, aero-tolerant Gram-negative spirochaete that exhibits a very broad mammalian host range^3^, and it is intimately associated with the colonic or caecal mucosa. AIS is a serious problem for the poultry industry, and infected chickens can produce wet and/or bloody faeces, diarrhoea and suffer from lower growth rate, lethargy and depression. There is also a late onset of egg laying, and egg numbers are reduced and of lower quality and often faecally contaminated^1,4,5^. Increased flock mortality can occur in severe cases of infection. PIS is characterised by diarrhoea and poor growth^2,5^. Humans with intestinal spirochaetosis may present with one or more non-specific clinical symptoms, e.g. abdominal pain, change in bowel habits, pseudo-appendicitis, irritable bowel, diverticulitis, chronic diarrhoea and rectal bleeding^6–10^. Zoonotic transmission to humans is associated with risk factors such as exposure to, and ingestion of, faecal contaminated water^5,11–13^, living rurally and often in close contact with animals, crowding conditions for both humans and animals, socioeconomic depression, travel to-and-from less economically developed countries, and positive HIV status^3^.

Both AIS and PIS are under-reported diseases with significant economic impact in food production, globally^1,5,14^. The economic costs of AIS and PIS have not been accurately assessed, though the financial loss to the UK poultry industry of AIS infection alone has been estimated to be £18 million per year^1^. Annual losses to both the poultry and porcine industries worldwide may be - speculatively by rough extrapolation - circa USD 4.7–6.0 billion/year for the former and USD 2–6 billion/year for the latter (Supplementary Information). There are no licensed vaccines to prevent Bp intestinal spirochaetosis, and control involves approaches such as good animal husbandry to reduce zoonotic transmission^3^, the potential use of oral probiotics, e.g. *Lactobacillus reuteri*^15^ and prebiotics and phytochemicals^16,17^, and the administration of antibiotics. Culling of afflicted flocks and herds and restocking with animals certified as Bp-free is a last-resort. Macrolides, lincosamides and the pleuromutilin tiamulin are currently used to treat AIS and PIS^1^, but resistance has been increasingly observed^18^. Current antibiotic therapies for HIS include co-amoxicillin and metronidazole^18^.

In the current study, we used a targeted computational approach to identify potential molecules that could be used to treat AIS and PIS, especially with increasing reports of the occurrence of resistance determinants in *B. pilosicoli*^19^. We decided to target the glutamate racemase enzyme for drug discovery^20^, since the enzyme is involved in the early phases of bacterial cell wall peptidoglycan biosynthesis^20–25^. The enzyme converts L-Glutamate to D-Glutamate and maintains a pool of amino acid for growth and incorporation into peptidoglycan, where it can provide the bacteria with a mechanism to evade host protease proteolytic cleavage. The amino acid sequences of the glutamate racemase from different organisms share several similarities in active site architecture and mechanisms of catalysis. Thus, we tested the hypothesis that a computational study of the *B. pilosicoli* glutamate racemase (abbreviated as Bp-MurI) coupled with high-throughput virtual screening of a compound library, in this case the Maybridge Library of > 51,000 Hit-like and Lead-like molecules, could identify potential inhibitors that could then be used to target the bacterium in vitro.

## Results and discussion

Intestinal spirochaetosis caused by the zoonotic spirochaete *Brachyspira pilosicoli* (Bp) is a significant problem for the poultry and swine industries. The absence of licensed vaccines and increasing bacterial resistance to antibiotics^1,3,18,19^ necessitates the development of new antimicrobial interventions. In the current study, we used a computational approach to identify potential small molecule inhibitors targeting the *B. pilosicoli*-glutamate racemase enzyme (Bp-MurI), which is pivotal for bacterial cell wall biosynthesis. The methodology involved protein modelling and validation to structural comparisons, ending with a reliable 3D structure of Bp-MurI.

### Protein modelling and validation

**Fig. 1:**
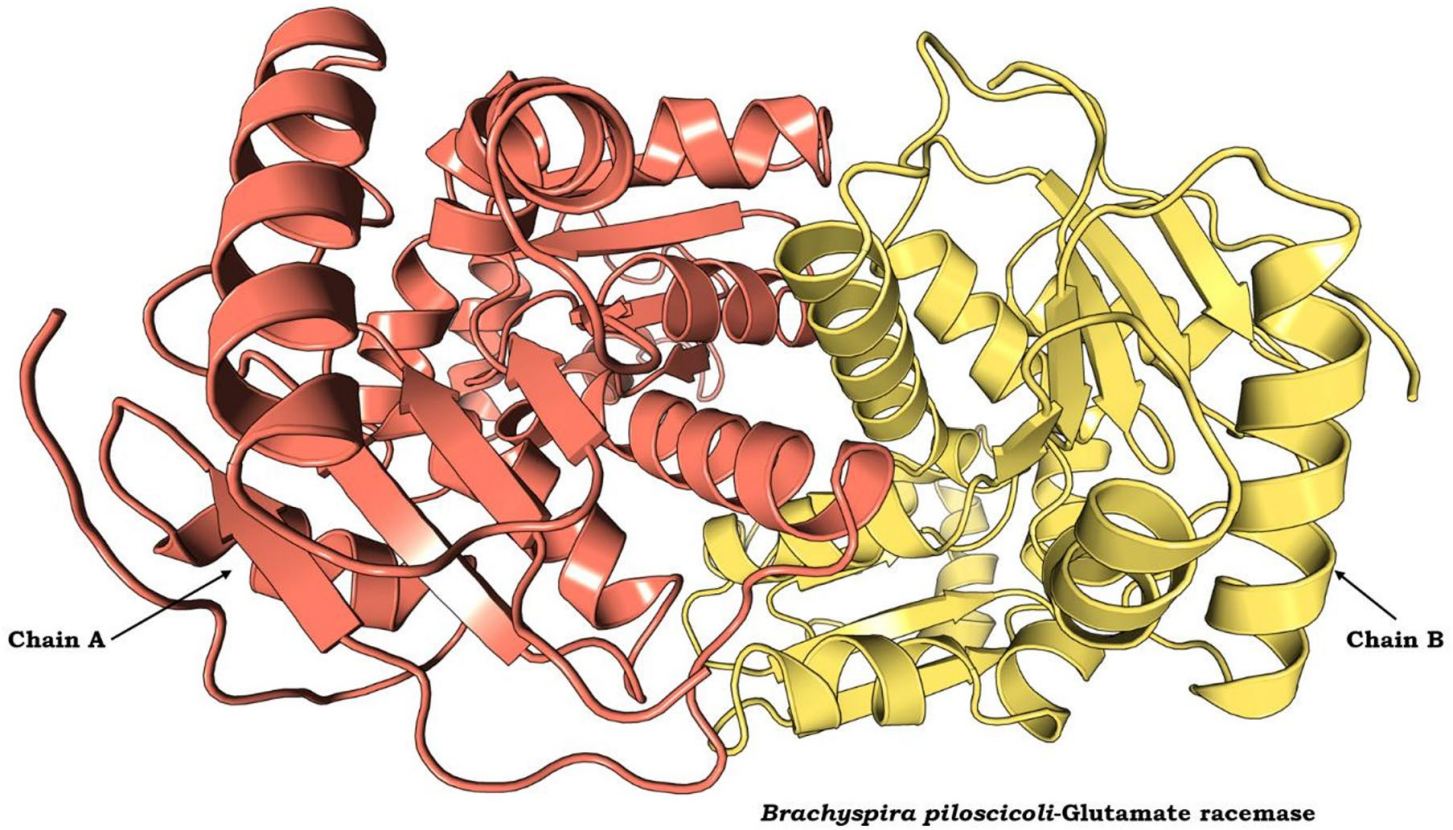
The 2JFQ template-based model of Bp-MurI generated by AlphaFold. The homo-dimeric protein consists of two identical chains (Chain A in brick red colour and Chain B in the yellow colour).

**Fig. 2:**
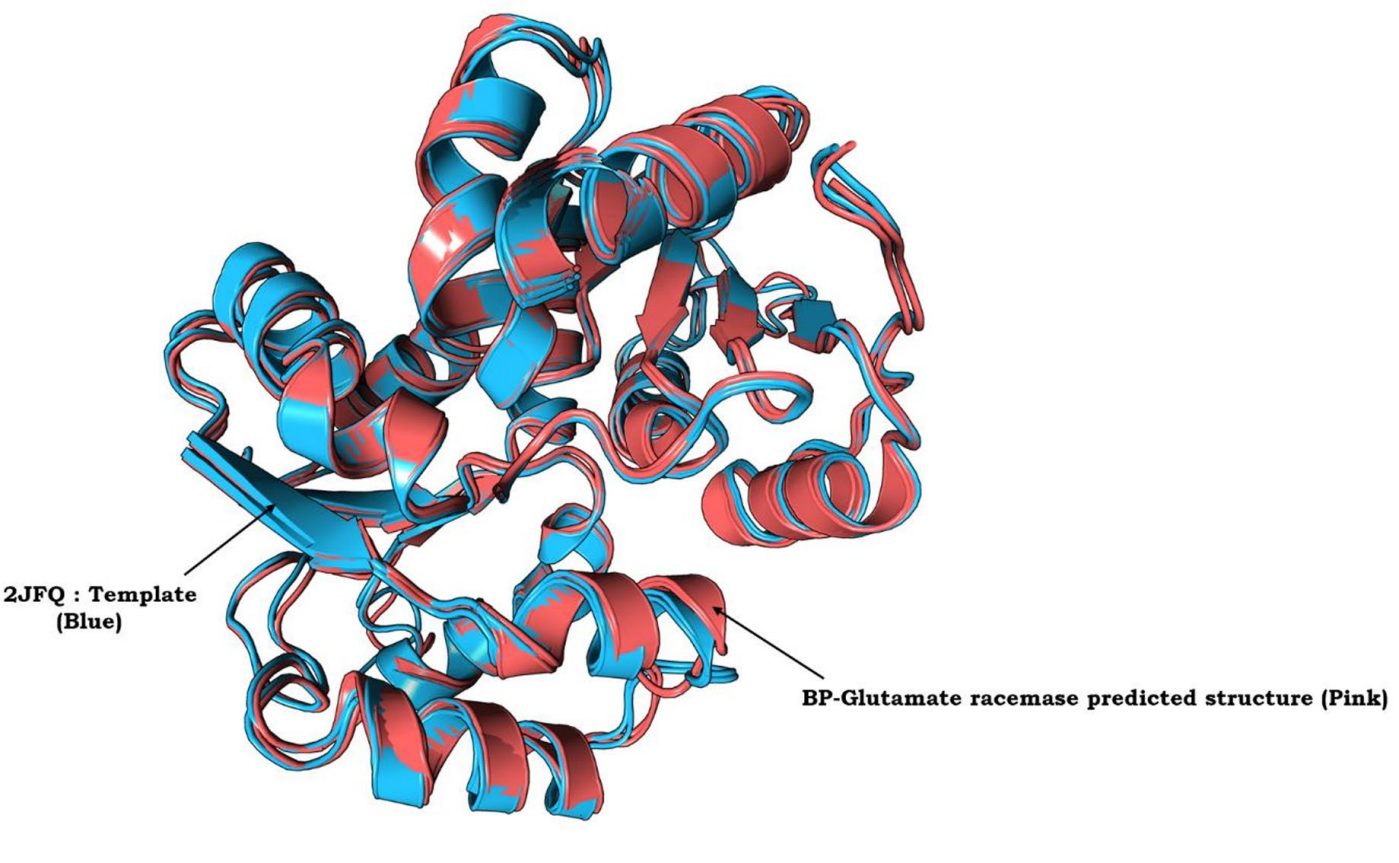
Superimposition of Template 2JFQ (in Blue) with Bp-MurI (in Pink). The superimposition shows the alignment between the crystal structure of *Staphylococcus aureus* glutamate racemase in complex with D-Glutamate (2JFQ, depicted in blue) and the homology-modeled structure of Bp-MurI (depicted in pink). The alignment was achieved using the SuperPose Version 1.0 server.

 In the absence of an experimentally derived crystal structure for Bp-MurI, we used homology modelling via AlphaFold to produce a 3D protein structure in silico. Structural PDB BLAST analyses revealed that the Bp-MurI sequence exhibited optimal alignment with the crystal structure of *S. aureus* glutamate racemase in complex with D-Glutamate (PDB Id: 2JFQ), with a query coverage of 99% and a sequence similarity of 41%. Notably, the gaps between the template and query proteins were minimal at 1%. The generated 3D structure of Bp-MurI describes a dimer composed of two homologous chains (Fig. 1). Superimposition of the modelled structure of Bp-MurI onto the 2JFQ template (Fig. 2), yielding a calculated RMSD value of 1.043 Å. This value underscores the substantial backbone similarity between the modelled protein and the template structure.

Further validation of the modelled structure was done using the PDBsum server and the Ramachandran plot (Supplementary Fig. 1). The data showed that 91.2% of residues resided in the allowed region, with an additional 8.4% falling into the ‘fairly’ favoured region, and 0.2% of residues located in the disallowed regions for Bp-MurI. These findings strongly endorsed the model’s overall stereo-chemical quality and stability. Additionally, an ERRAT analysis with the SAVES v6.0 server, examined the overall quality of the model, and revealed an overall quality factor of 98.43 (Supplementary Fig. 2). Collectively, this suite of assessments established the model’s backbone conformation, non-bonded interactions, and energy scores well within the parameters that indicated a high-quality and reliable structural model.

### Active site analysis and validation

**Fig. 3:**
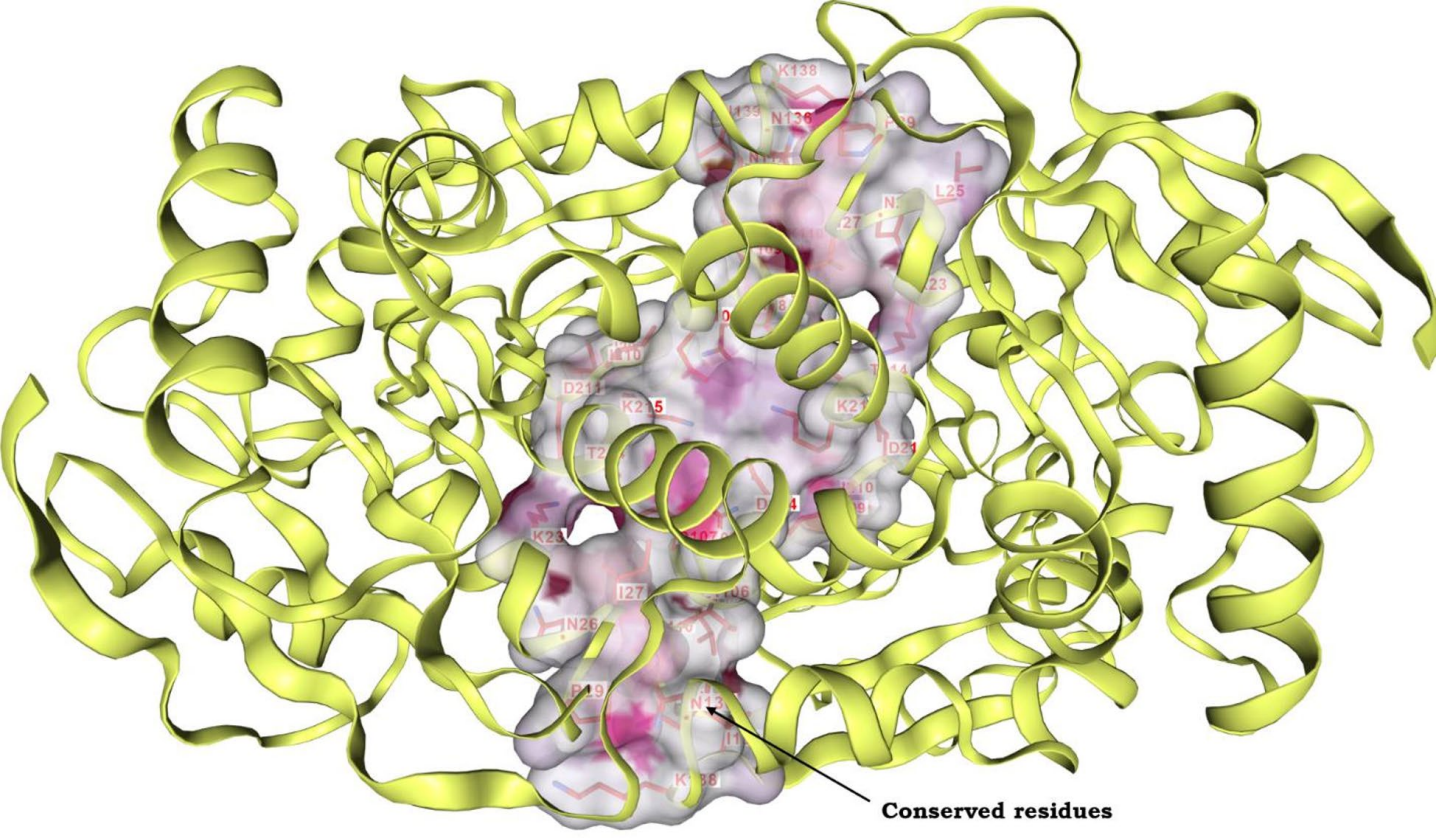
The binding pocket (active site) predicted by DeepSite for the modelled Bp-MurI protein. The active site regions revealed conserved residues crucial for protein-ligand binding, preserving the architectural integrity of the binding cavity.

The delineation of potential ligand-binding sites offered insights into the active site landscape and revealed the network of interactions between the protein and ligands, which is required knowledge for subsequent structure-based virtual screening of compound libraries. We examined the active site architecture of the modelled Bp-MurI protein using DeepSite. Exploring the binding cavity within Bp-MurI revealed a predilection for amino acid residues within the cleft between the enzyme’s two domains. As shown in Fig. 3, the main constituents of the binding site included residues from Chain A (LYS23, ASN26, ILE27, ASP104, ASP107, ASN108, ASN208, ILE209, ILE210, ASP211, THR214, LYS215) and Chain B (ASP104, ILE106, ASP107, ASN108, THR109, LYS110, ASN112, ASN136, ILE139, LYS215). The conserved nature of these active site residues imparted confidence in the reliability of our model.

**Fig. 4:**
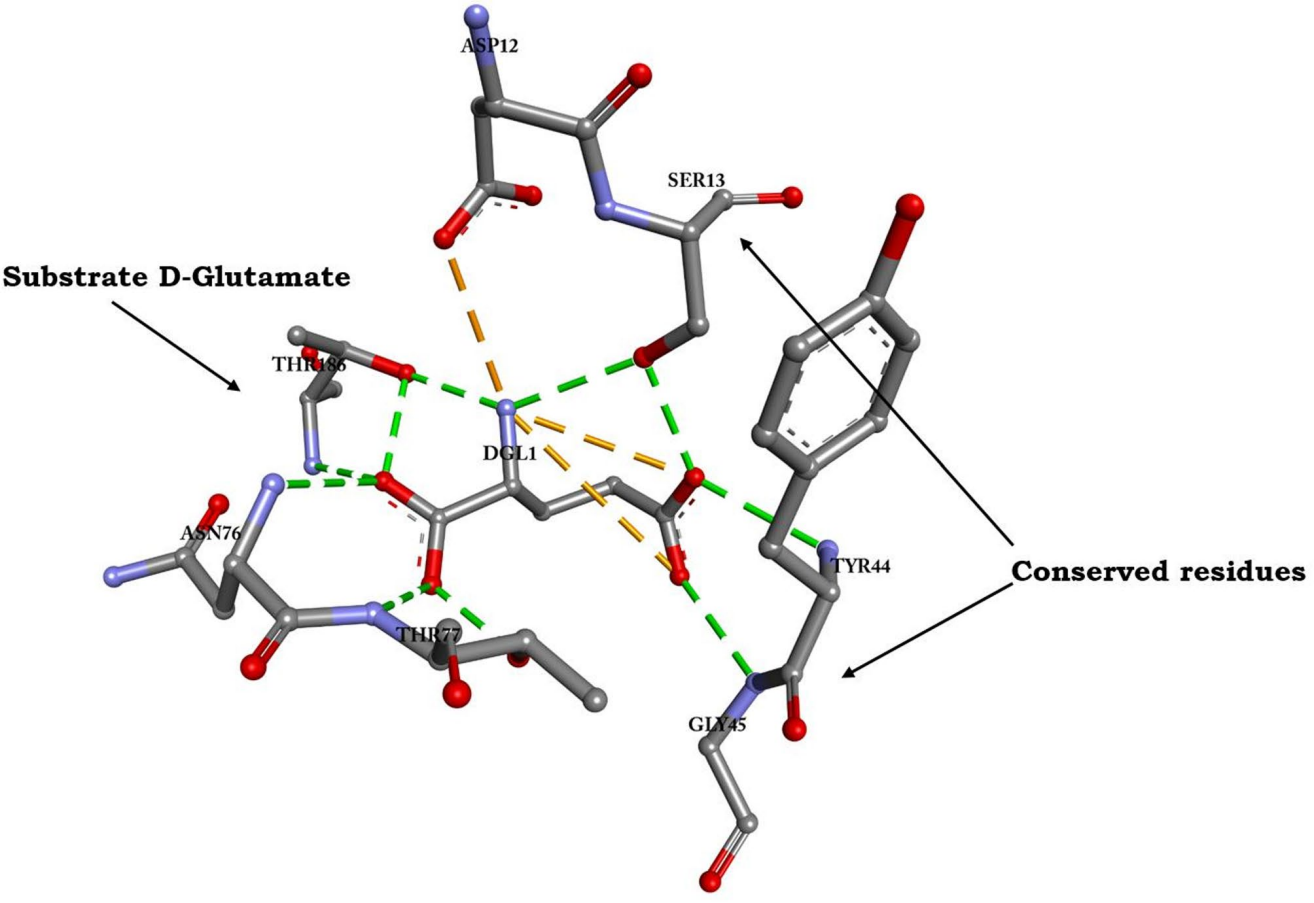
Molecular interactions between the substrate D-Glutamate and active site residues in the Bp-MurI protein. The figure shows the interplay and specific interactions occurring at the binding site between the protein and its substrate.

Validation extended beyond intrinsic assessments, and our findings were corroborated with existing literature on glutamate racemases from pathogens like *N. gonorrhoeae* and *Mycobacterium tuberculosis*^22,26^. Referencing these studies, we completed targeted docking exercises and reconstructed the binding cavity using the same residues on the protein surface. This approach affirmed the accuracy of our predicted binding site, which was consistent with the published literature. The molecular interactions of the substrate D-Glutamate in proximity to the identified binding site residues in Bp-MurI are shown in Fig. 4.

### Pharmacophore generation

**Fig. 5:**
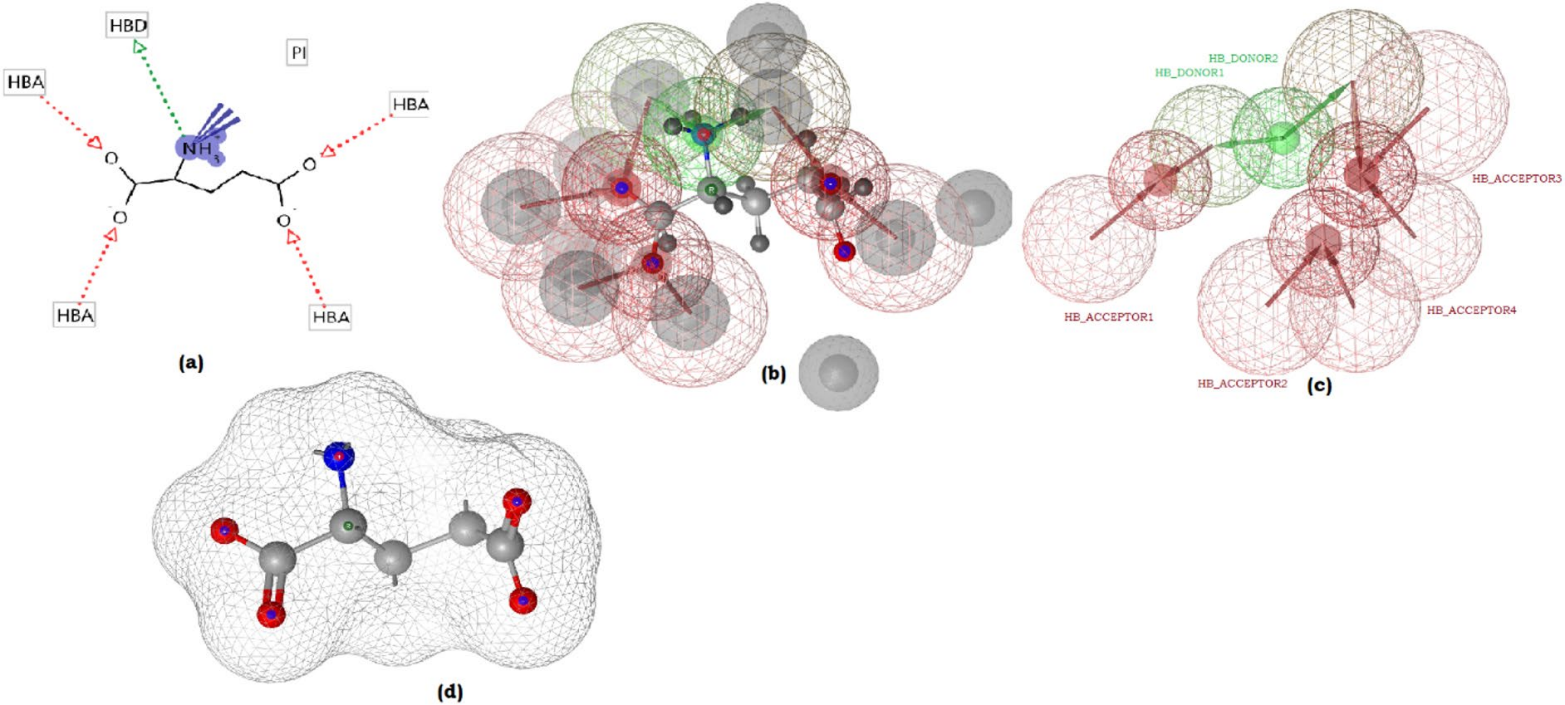
Generation of pharmacophores from D-Glutamate binding to Bp-MurI. (**a**) Receptor enzyme glutamate racemase showing interactions with the catalytic site residues of the predicted model. (**b**) Visualisation of the structure-based pharmacophore model generated using the optimal docking pose, revealing key features governing ligand recognition. (**c**) Mapping of distinct features within the pharmacophore model, including hydrogen bond donors, hydrogen bond acceptors, and hydrophobic regions. (**d**) Surface view of the D-Glutamate ligand within the active site, providing a detailed perspective of its spatial arrangement.

A pharmacophore displays abstract molecular features that are vital for ligand recognition by a biological macromolecule^27^. In the current study, we elucidated the pharmacophore requirements for the ligand D-Glutamate binding to the receptor Bp-MurI, using a structure-based pharmacophore generation approach. D-Glutamate was docked into the conserved binding site of the modelled Bp-MurI receptor, and the optimal docking pose of D-Glutamate was selected based on the highest docking score and the lowest RMSD regarding D-Glutamate. The ‘receptor-ligand pharmacophore generation module’ of Biovia DS 2020 facilitated the creation of the structure-based pharmacophore^28,29^. This tool interprets ligand-receptor interactions based on key pharmacophore features, including hydrogen bond donors, hydrogen bond acceptors, hydrophobic, and hydrophilic regions. Additionally, an ‘excluded volume’ was incorporated into the active site to ensure the maintenance of the steric circumference of the macromolecule, enhancing the identification of an optimal pharmacophore^30^. Selection of the optimal pharmacophore model was guided by evaluating the selectivity scores and the number of features. Among the candidate models, one emerged as the most promising, characterised by six features - four hydrogen bond acceptors and two hydrogen bond donors (AAAADD i.e. Pharm1). Pharmacophore Pharm1 exhibited a selectivity score of 9.4218, indicative of its capability to capture the essential features governing ligand recognition within the binding site. Visual representation of the finalised pharmacophore Pharm1, with a view of the spatial arrangement and characteristics of the identified features essential for molecular recognition, is shown in Fig. 5.

The finalised pharmacophore model, with its well-defined AAAADD configuration, encapsulated the critical molecular characteristics for effective binding of D-Glutamate to the Bp-MurI active site. The selectivity score underscored the model’s discriminatory power, emphasising its capacity to distinguish ligands with precision. Moreover, the generated pharmacophore served as a blueprint for understanding the intricate ligand-receptor interactions and for HTVS to identify potential drug candidates.

### HTVS

The Maybridge library (Supplementary Dataset 1) was selected for virtual screening due to its high chemical diversity, drug-like properties, and suitability for early-stage small molecule discovery studies. Compared to other libraries like ZINC, which includes a wide range of unfiltered or non-drug-like molecules, and DrugBank, which focuses on approved or investigational drugs, Maybridge offers a curated collection of lead-like compounds optimised for Hit identification and experimental follow-up. Furthermore, these compounds are available commercially without restriction.

A final number of compounds from the Maybridge High Throughput Screening (HTS) Library of > 51,000 drug-like compounds that could be possible Hit compounds for Bp-MurI were identified using a virtual screening technique. This HTVS protocol is based on the application of sequential filters to narrow down a pool of compounds from a dataset library to a final selection of Hit compounds. In our study, the Pharm1 pharmacophore model (with features: AAAADD) was subjected to virtual screening of the Maybridge HTS Library. The ‘Build 3D database’ module and ‘Screen library’ module of Biovia DS 2020 were used to create and screen the data set of the library. The cutoff value of 2.0 was chosen concerning the fit value of our positive control ligand i.e. D-Glutamate. After the initial round of screening based on pharmacophore mapping, 4,110 compounds were shortlisted and mapped successfully against Pharm1 (AAAADD) and were sorted based on their fit values. The pharmacophore fit value is typically displayed as the geometric fit of features to the 3D-structure-based pharmacophore model. The molecules with the highest fit score in the validated pharmacophore model should, theoretically, interact with Bp-MurI. Therefore, 3,050 Hit compounds were chosen that showed a fit value of more than 2.0. Next, 2,569 Hit compounds were filtered using ADMET, TOPKAT, VEBER, and Lipinski’s filter and were able to pass all the pharmacokinetic parameters and were subjected to docking studies. This screening pipeline is represented in our graphical abstract.

During our initial HTVS, we used targeted docking and focused on the precise pocket predicted as the binding site. Using this approach, we shortlisted ten small molecule inhibitors as Hit compounds, based on their significant docking scores and fit values (Table 1). To thoroughly explore potential allosteric sites on the modeled protein surface, we complemented this strategy with blind docking. In this screening, compound Hit 2 emerged as noteworthy, since it docked at a site different from the initially predicted binding pocket. On closer inspection, it became evident that the spatial interactions at this alternative site were suboptimal and deviated from the expected behavior. Thus, as part of our analysis and refinement process, we excluded Hit 2 from further consideration and moved forwards with compounds Hit 1, Hit 3, and Hit 4. This decision was based on their favorable interactions within the predicted binding pocket, a critical aspect for their potential as effective small molecule inhibitors. Hit 2, while promising, was excluded due to its failure to engage optimally with crucial binding site residues. This screening and selection process ensured that only the most promising compounds proceeded to the subsequent stages of our investigation.

**Table 1:** Top ten Hit compounds shortlisted from High-Throughput Virtual Screening of the Maybridge HTS Library containing > 51,000 compounds.

S. No.	Compound ID	Catalogue Ref.	Compound Name	-CDOCKER Energy	Fit Value	Mol. Wt.	Toxicity	SMILES
Hit 1	HTS02114	HTS02114SC	Unnamed Molecule12127	44.0037	2.14099	497.613	Non-Mutagen	O = C(OC1 = CC(C(F)(F)F)=NN1C)C(C(C2 = C(Cl)C = CC=C2Cl)=NO3)=C3C
Hit 2	AW00689	AW00689SC	Unnamed Molecule979	41.7663	2.86161	356.346	Non-Mutagen	COC1 = C(OC)C = CC(NC(NC2 = C(Cl)C = CC=C2) = S)=C1
Hit 3	AW00718	AW00718SC	Unnamed Molecule962	41.0694	2.88285	379.384	Non-Mutagen	BrC1 = CC(C(N = NC(SCC2 = CC = C(C)C = C2)=N3)=C3N4)=C4C=C1
Hit 4	HTS04172	HTS04172SC	Unnamed Molecule43708	40.6653	2.37424	433.468	Non-Mutagen	N#CC1 = C(NC(CN2CCN(C3 = CC = CC=C3)CC2) = O)SC4 = C1CCC4
Hit 5	AW00694	AW00694SC	Unnamed Molecule950	39.0649	2.90517	390.403	Non-Mutagen	ClC1 = C(SC2 = C1C = CC=C2)C(OC3 = O)=NC4 = C3C = CC(Cl)=C4
Hit 6	AW01216	AW01216SC	Unnamed Molecule2370	39.0622	2.23123	454.589	Non-Mutagen	O = C(N/N = C(C(OC) = O)\CC(OC) = O)C1 = CC(C(F)(F)F) = CC(C(F)(F)F)=C1
Hit 7	HTS10007	HTS10007SC	Unnamed Molecule34186	38.3345	2.83712	439.538	Non-Mutagen	O = S(C1 = CC = C(Cl)C = C1)(CC(O)COC(C = C2) = CC=C2Cl) = O
Hit 8	AW00696	AW00696SC	Unnamed Molecule960	38.0711	2.71032	372.413	Non-Mutagen	COC1 = CC = CC(C=C2C#N)=C1OC2 = N
Hit 9	AW00936	AW00936SC	Unnamed Molecule1446	37.5307	2.18101	531.502	Non-Mutagen	O = S(C1 = CC = C(C)C = C1)(NNC(NCC2 = CC = CC=C2) = S) = O
Hit 10	KM08643	KM08643SC	Unnamed Molecule24603	36.7855	2.45819	496.46	Non-Mutagen	BrC1 = C(NC(C(Cl)(Cl)Cl) = O)N(N=C1C)C2 = CC = CC=C2

Hit 1 = methyl 5-({[(1,3-dibenzylhexahydro-5-pyrimidinyl)methyl]amino}sulfonyl)-2-methyl-3-furoate; Hit 3 = 1-(1 H-indol-1-yl)-3-[(2-{[4-(trifluoromethyl)pyrimidin-2-yl]amino}ethyl)amino]propan-2-ol; Hit 4 = 5-({2-[(4-methylphenyl)sulfonyl]ethyl}sulfanyl)-4-[2-(trifluoromethyl)phenyl]-4 H-1,2,4-triazol-3-ol.

### Molecular docking (MD)

**Fig. 6:**
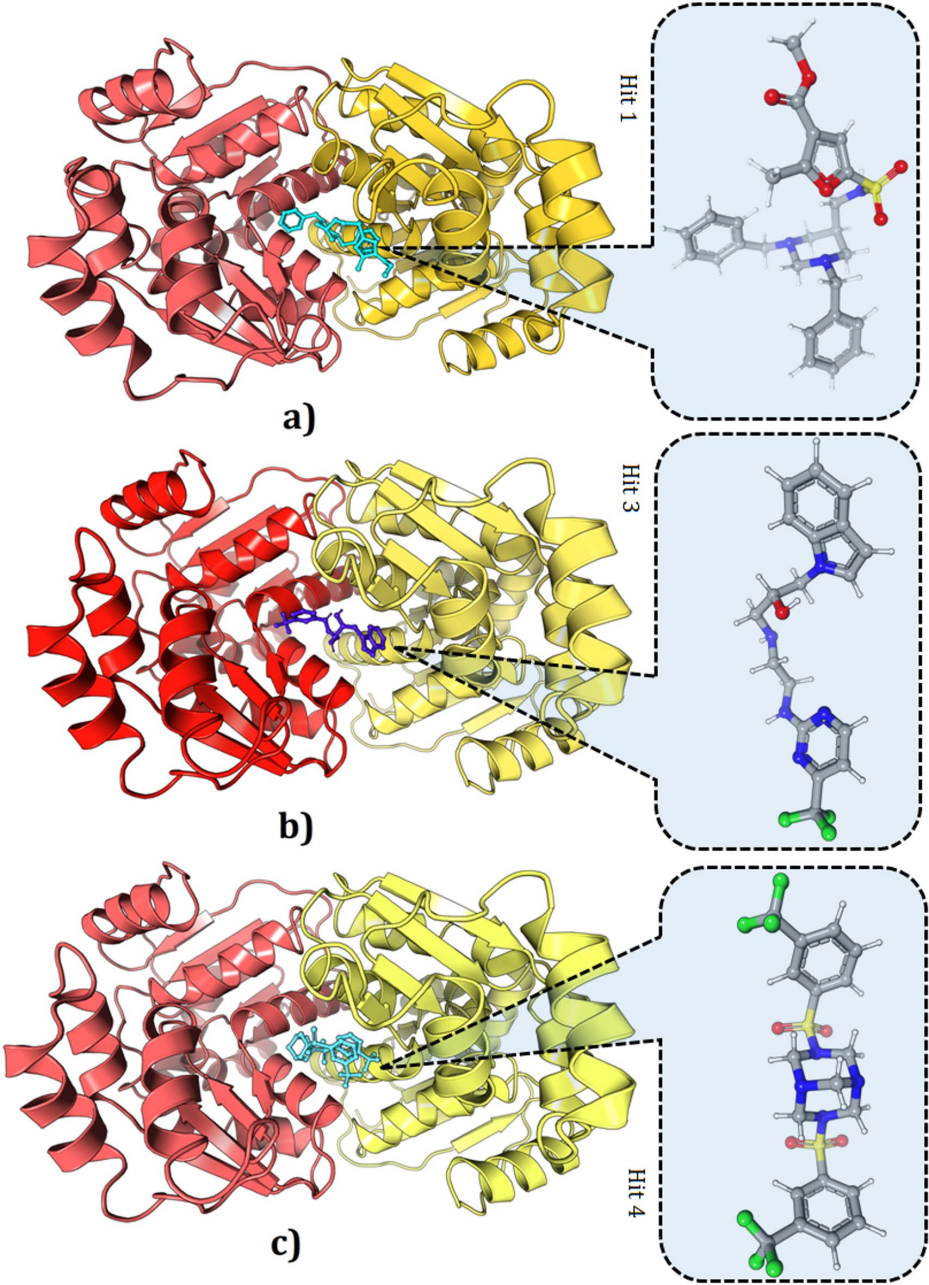
Molecular Docking of Bp-MurI with compound **(A) Hit 1.** Visualisation of the molecular docking interaction between Bp-MurI (Red and Yellow chains) and Compound Hit 1 (green), providing insights into the spatial arrangement within the protein’s predicted binding pocket. **(B) Hit 3.** Visualisation of the molecular docking interaction between Bp-MurI (Red and Yellow chains) and Compound 3 (blue), providing insights into the spatial arrangement within the protein’s predicted binding pocket. **(C) Hit 4.** Visualisation of the molecular docking interaction between Bp-MurI (Red and Yellow chains) and Compound 4 (green), providing insights into the spatial arrangement within the protein’s predicted binding pocket.

MD is a vital step in the drug design process^31^ and was used to assess the binding ability of the Hit compounds to Bp-MurI. The 2569 Hit compounds obtained above were docked with the modelled Bp-MurI using the CDOCKER tool of Biovia DS 2020 to evaluate their binding capacity, which satisfied the pharmacophore model’s characteristics. We successfully docked 2569 compounds, and from these, 100 top Hit compounds exhibited favorable binding interactions with Bp-MurI. This set of 100 compounds included the 10 Hit compounds in Table 1. Based on their structural and biological properties, molecular docking for Hit 1, Hit 3 and Hit 4 compounds with Bp-MurI is shown in Fig. 6A, B and C, respectively. These final three Hit compounds were shortlisted and analysed further, based on their earlier known biological activity, interaction with active site residues as well as other docking parameters. Although classical docking validation approaches such as benchmarking against known inhibitors were not feasible due to the limited availability of experimentally validated MurI inhibitors for *B. pilosicoli*, the integration of substrate-guided docking, pharmacophore filtering, and molecular dynamics simulations provided a multi-layer validation framework supporting the reliability of the screening pipeline.

The selection of Hit 1, Hit 3 and Hit 4 was based on their favorable docking scores and fit values obtained during the virtual screening process. The CDOCKER Energy scores and Fit Values serve as key metrics for evaluating the binding affinity and overall fitness of the compounds within the binding site^32^. The superior docking scores and fit values of the three compounds suggested strong binding affinities and optimal spatial orientations within the predicted binding pocket. These scores indicated the potential for crucial interactions with the target protein, making them candidates for further investigation and development as small molecule inhibitors. Hit 1, -3 and − 4 compounds showed good interactions with some of the substrate-binding amino acids, such as Chain A LYS23, ASP104, ASP107, ASN108, ASN208, ILE209, ILE210, ASP211, THR214 and LYS215, and Chain B ASP104, ASP107, ASN108, ILE209 and LYS215, and they fit well within the active site cavity of the protein. The docking scores (CDOCKER Energy), chemical structures, Fit values and important interactions of the three Hit compounds are provided in Table 2.

**Fig. 7:**
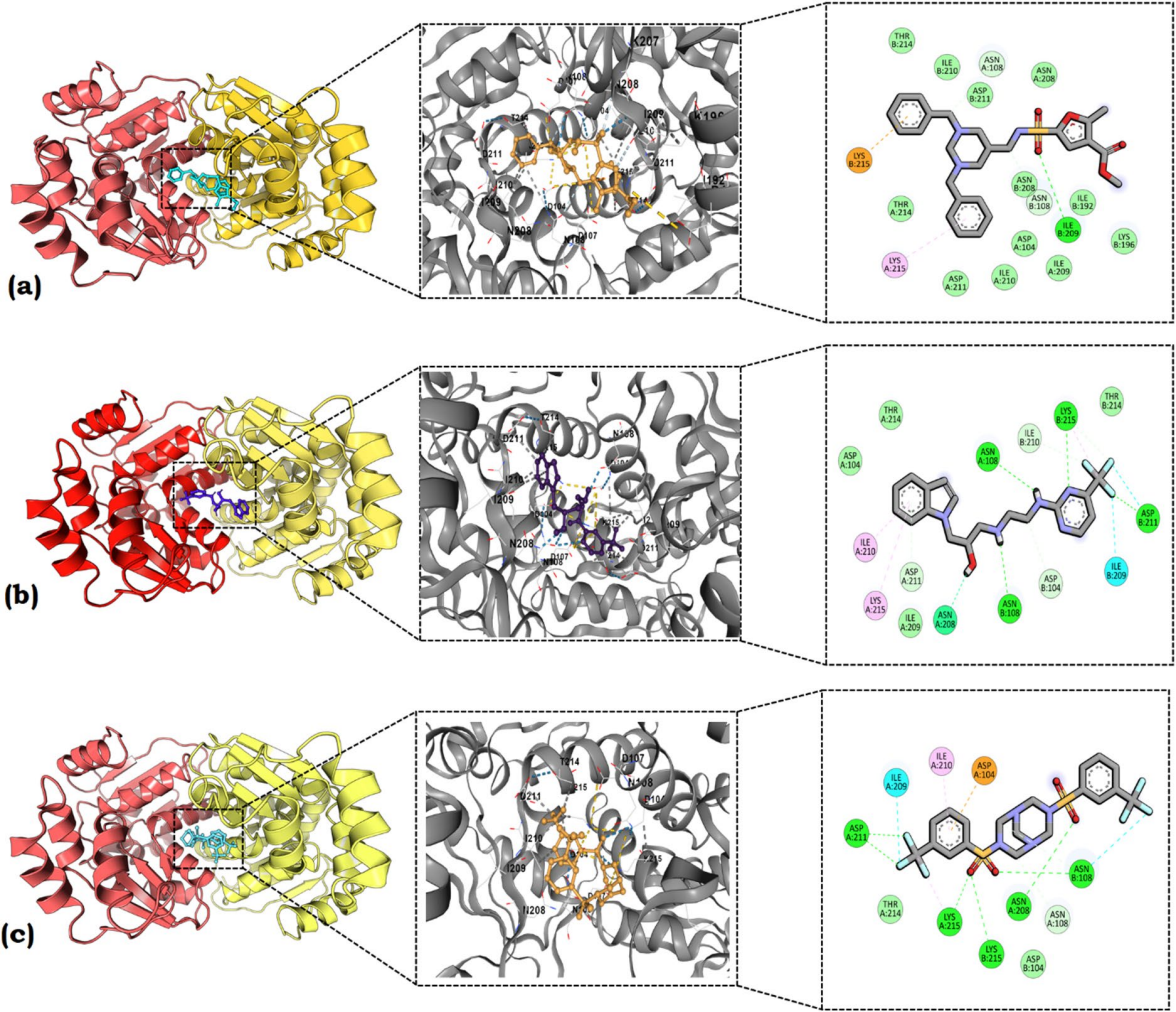
Docking Analysis of top three Hit compounds with Bp-MurI binding cavity with 2D interaction diagrams. Molecular docking, the enlarged interaction pattern and 2D interaction diagrams for (a) Hit 1 (b) Hit 3 and (c) Hit 4 are shown. This figure presents the collective molecular docking scenarios for the three compounds within the binding cavity of Bp-MurI. Notably, all docked ligands demonstrate optimum docking scores, reinforcing their strong affinity, while consistently retaining interactions with conserved residues critical for protein-ligand binding interactions.

In the present study, we carried out an MD analysis to evaluate the binding interactions of the top three Hit compounds with Bp-MurI. The results are depicted in Fig. 7, which provides an in-depth view of the MD scenarios, enlarged interaction patterns, and 2D interaction diagrams for each of the shortlisted compounds. There was a consistent retention of interactions with conserved residues within the binding cavity. This observation is crucial as it highlighted the compounds’ abilities to engage with key residues essential for protein-ligand binding interactions. The preservation of such interactions further substantiates the potential therapeutic relevance of the identified Hit compounds^33^.

**Table 2:** Summary of the characteristics of the top three Hit compounds shortlisted from HTVS.

S. No	Compound Name	Chemical Structure	-CDOCKER Energy	Fit Value	Crucial residues involved
**Hit 1**	HTS02114		44.0037	2.14099	**Chain A** LYS23, ASP104, ASP107, ASN108, LYS110, ILE192, LYS196, LYS207, ASN208, ILE209, ILE210, ASP211, THR214, LYS215 **Chain B** LYS23, ASP104, ASP107, ASN108, LYS110, ILE209, ASP211, THR214, LYS215
**Hit 3**	AW00718		41.0694	2.88285	**Chain A** LYS23, ASP104, ASN108, ILE192, ASN208, ILE209, ILE210, ASP211, THR214, LYS215 **Chain B** ASP104, ASP107, ASN108, LYS215
**Hit 4**	HTS04172		40.6653	2.37424	**Chain A** ASP104, ASP107, ASN108, ASN208, ILE209, ILE210, ASP211, THR214, LYS215 **Chain B** ASP104, ASP107, ASN108, ILE209, LYS215

The chemical structures, fit values and CDOCKER energies are reported as well as the crucial residues of the enzyme involved in the interactions with the compounds.

### Decoding protein-ligand interactions: the crucial residues involved and the hydrophobicity landscape in the binding pocket of Bp-MurI

The investigation into the molecular interactions between Bp-MurI and Hit 1, Hit 3 and Hit 4 compounds revealed a network of crucial residues that maintained the binding interactions. The identification of these residues offered insights into the specific amino acids contributing to the stability and affinity of the protein-ligand complexes (Table 2). The interaction profile of Hit 1 demonstrated extensive involvement of residues from both Chain A and Chain B. Notably, these residues contributed to hydrogen bonding and hydrophobic interactions, ensuring a robust binding interface. The spatial arrangement of these residues within the active site demonstrated an inclusive engagement of the ligand with the protein. Hit 3 exhibited a distinct set of interacting residues, forming hydrogen bonds and establishing pi-stacking interactions within the binding pocket. The hydrophobicity analysis suggested a balanced distribution of hydrophilic and hydrophobic residues, contributing to the overall stability of the complex. Hit 4 engaged with a set of key residues involved in hydrogen bonding and forming a salt bridge, enhancing the electrostatic interactions within the binding site (Table 2).

**Fig. 8:**
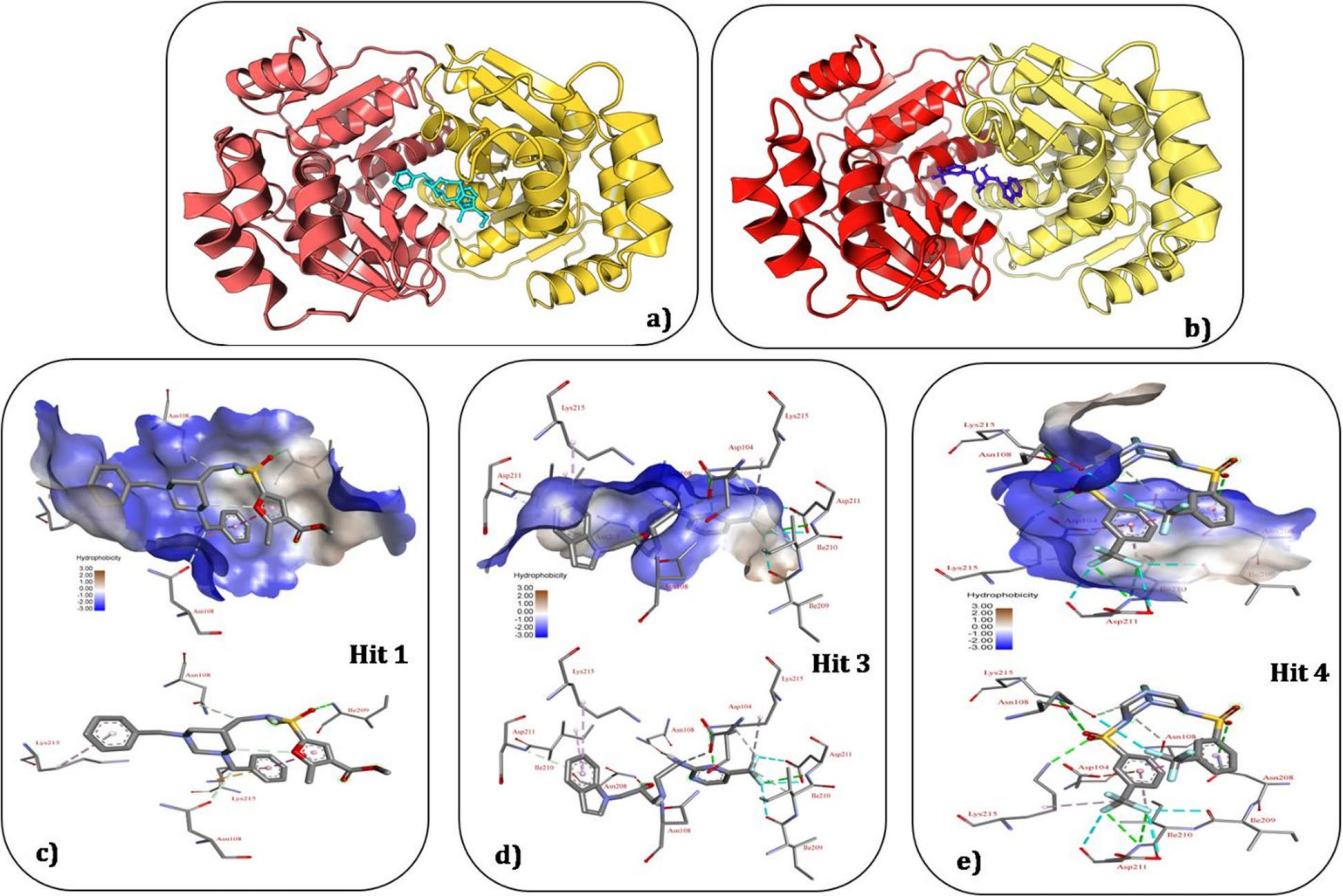
Hydrophobicity analysis of the Bp-MurI binding pocket (**a-b**) highlighting the distribution of hydrophobic (yellow) and hydrophilic (blue) residues involved in the interaction network (**c-e**). The profile reveals a dynamic interplay between hydrophobic and hydrophilic amino acids contributing to ligand binding stability.

The hydrophobicity analysis underscored the prevalence of hydrophobic residues in the interaction network (Fig. 8) and profiling of the binding pocket revealed a dynamic interplay between hydrophilic and hydrophobic residues. Amino acids with highly negative values, such as ASP and ASN, tended to form hydrogen bonds with water, whereas hydrophobic residues with positive values, including ILE and LYS, contributed to the formation of a stable hydrophobic core within the protein interior. The identified crucial residues would appear to play a role in mediating the interactions between Bp-MurI and the Hit compounds, influencing the binding affinity and stability of the complexes. The diverse nature of interactions, ranging from hydrogen bonding to pi-stacking and salt bridges, highlighted the complexity of the binding events.

### Molecular dynamics simulations (MDS)

To elucidate the dynamic behaviour and stability of the Bp-MurI-ligand complexes, MDS were done for 100 ns on compounds Hit 1, Hit 3, and Hit 4 complexed with Bp-MurI, alongside the unbound Bp-MurI control. These simulations discerned conformational stability, protein-ligand interactions, and compactness of the protein structure upon ligand binding, contributing to an understanding of the impact of each compound on the stability of Bp-MurI.

**Fig. 9:**
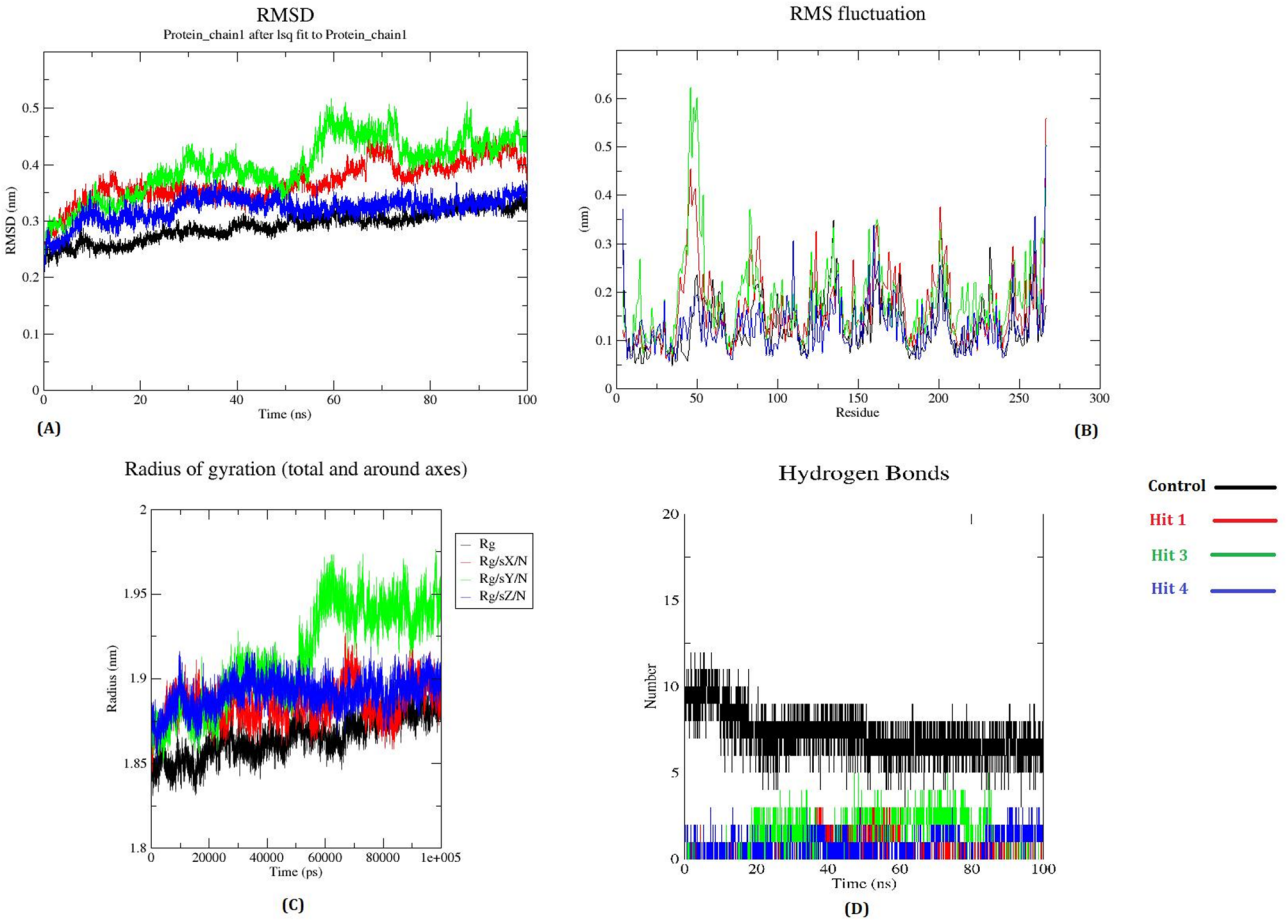
Plots to investigate the energy conformations, stability, and fluctuations of Bp-Rac and Hit compounds in the bound state: (**A**) RMSD, (**B**) RMS fluctuations, (**C**) radius of gyration, and (**D**) hydrogen bonds. The black colour shows the control i.e. Bp-Rac-glutamate only (unbound state) whereas the red, green and blue colours represent Bp-Rac bound to Hit 1, Hit 3and Hit 4 respectively.

1. Root Mean Square Deviation (RMSD). The RMSD values assessed the overall structural stability of the Bp-MurI-ligand complexes throughout the 100 ns simulation (Fig. 9A). All three complexes, as well as the unbound control, exhibited stable RMSD values around 0.2 nm, indicating consistent conformational stability during the simulation period. Bp-MurI-Hit 3 complex showed slightly higher deviations in comparison to the other complexes, and Bp-MurI-Hit 4 complex displayed minimal fluctuations, suggesting potentially a more robust and stable interaction with the protein. The unbound control maintained comparable stability, underscoring the consistency of the protein’s inherent structural conformation, with ligand binding notably stabilising the structure in specific cases.

ii) Root Mean Square Fluctuation (RMSF): RMSF analysis explored the flexibility of individual amino acid residues upon ligand binding (Fig. 9B). Notable fluctuations were observed in the 28–65 residue region in both the Bp-MurI-Hit 1 and Bp-MurI-Hit 3 complexes, indicating flexibility in this region upon binding. Conversely, the Bp-MurI-Hit 4 complex exhibited minimal fluctuations in these same regions, pointing to a stabilising effect of Hit 4 on the more flexible parts of the protein. The reduced fluctuations observed in Bp-MurI-Hit 4 suggested that this compound induced greater rigidity within the protein, enhancing its structural stability upon binding.
III) Radius of Gyration (Rg): To investigate changes in the protein’s compactness and folding behaviour, Rg values were calculated across all complexes and the control (Fig. 9C). The average Rg values for the Bp-MurI complexes ranged between 1.83 nm and 1.87 nm, indicating that the protein maintained its compactness throughout the MD simulations. Bp-MurI-Hit 1 and Bp-MurI-Hit 4 complexes displayed stabilised Rg values after approximately 20 ns, reflecting increased compactness and suggesting a more stable folding behaviour of the enzyme upon ligand binding. The unbound control showed a similar range of Rg values, indicating that ligand binding had a measurable impact on protein folding dynamics, particularly for Hit 1 and Hit 4 compounds. Bp-MurI–Hit 3 complex also maintained an average Rg value within this narrow range, although with minor fluctuations throughout the simulation, suggesting that the compound preserved overall protein compactness without inducing significant folding perturbations.
IV) Hydrogen Bond Analysis: Hydrogen bonds play a critical role in stabilising protein-ligand interactions^34^. Throughout the 100 ns simulation, the number of hydrogen bonds between Bp-MurI and each of the ligands was monitored (Fig. 9D). The unbound Bp-MurI control formed the highest number of intramolecular hydrogen bonds, reflecting its more compact and internally stabilized conformation. Ligand binding reduced the total number of intramolecular hydrogen bonds, consistent with conformational adjustments required to accommodate the ligand within the active site. The consistency and strength of hydrogen bonding in the Bp-MurI-Hit 4 complex further highlighted its potentially superior binding interaction. By contrast, the Bp-MurI–Hit 3 complex consistently formed a moderate number of hydrogen bonds during the trajectory; whilst fewer in number compared to Hit 4, these bonds were stable across the simulation, which may partly explain the biological activity of Hit 3 observed in vitro below.

### MM-PBSA binding free-energy calculations

MM-PBSA calculations were done to quantify the thermodynamic stability of Bp-MurI complexes and complement the docking and MD analyses. The averaged binding energy components across 200 snapshots for each ligand-bound system and the unbound control are summarised in Table 3.

**Table 3:** MM-PBSA binding free-energy profile (kJ/mol) of Bp-MurI complexes.

Complex	ΔE_vdw	ΔE_elec	ΔG_solv-PB	ΔG_solv-NP	ΔG_bind
Control	-134.09	-169.67	248.38	-19.11	-319.03
Hit 1	-118.01	-131.76	279.25	-21.11	-378.21
Hit 3	-144.39	-176.37	310.00	-26.89	-401.26
Hit 4	-152.27	-198.20	354.00	-39.19	-428.11

**Fig. 10:**
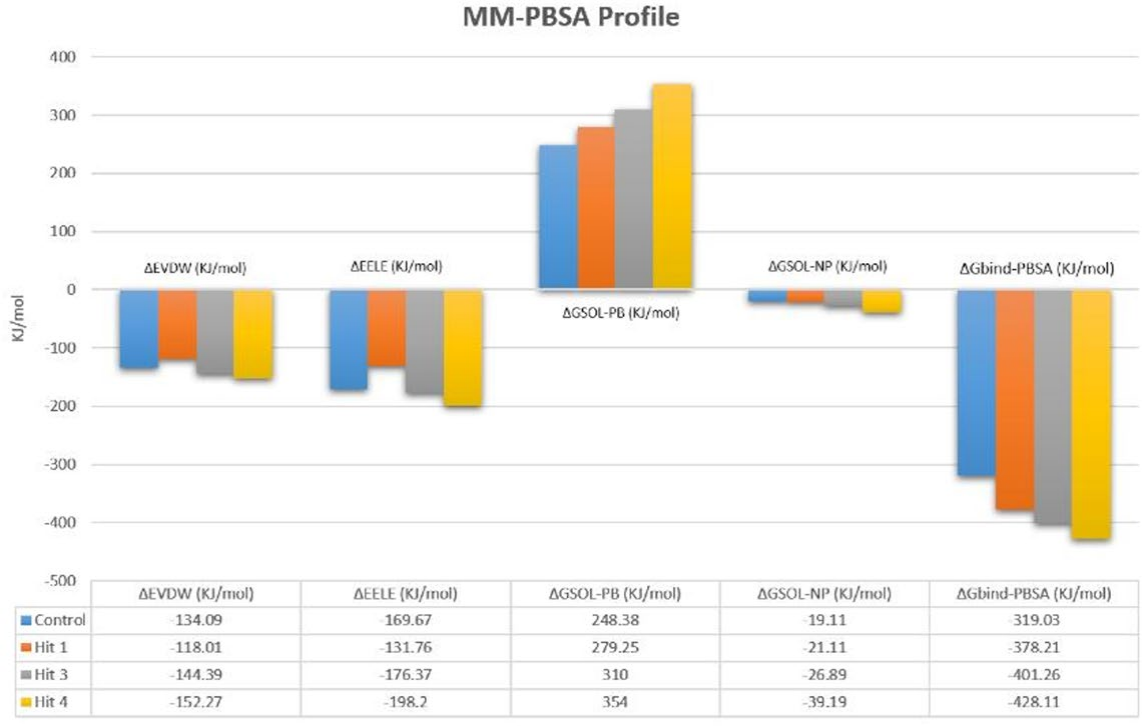
MM-PBSA binding free-energy profile of Bp-MurI complexes. MM-PBSA energy decomposition showing van der Waals, electrostatic, polar solvation, nonpolar solvation, and total binding free energies for the unbound protein (Blue) and ligand-bound complexes (Hit 1 Orange, Hit 3 Grey, Hit 4 Yellow). Values represent averages from the final 20 ns of the MD simulation. Hit 4 showed the most favourable predicted ΔG_bind, followed by Hit 3 and Hit 1.

The MM-PBSA analysis revealed that all three hits demonstrated more favourable binding energies compared to the unbound protein, supporting their predicted affinity for the Bp-MurI active site. Among the tested molecules, Hit 4 exhibited the strongest predicted binding (ΔG_bind = − 428.11 kJ/mol), followed by Hit 3 (–401.26 kJ/mol), while Hit 1 demonstrated the lowest affinity among the active compounds. The dominant energetic contributors were van der Waals and electrostatic interactions, while polar solvation energy opposed binding, as expected for ligands binding to a charged catalytic pocket. A bar-graph representation of these components is shown in Fig. 10.

Notably, although Hit 4 exhibited the most favourable predicted binding free energy, it did not demonstrate antimicrobial activity in vitro. This apparent discrepancy highlights a key limitation of binding energy-based predictions, as strong target affinity does not necessarily translate into whole-cell efficacy. Factors such as limited permeability across the Gram-negative outer membrane, active efflux, and compound sequestration in complex growth media may reduce intracellular bioavailability. In addition, MM-PBSA reflects thermodynamic binding stability but does not capture functional inhibition or catalytic interference. In contrast, Hit 3 demonstrated both favourable binding energetics and measurable biological activity, suggesting a more optimal balance between target engagement and cellular accessibility.

### Activity of hit compounds against B. pilosicoli in vitro

**Fig. 11:**
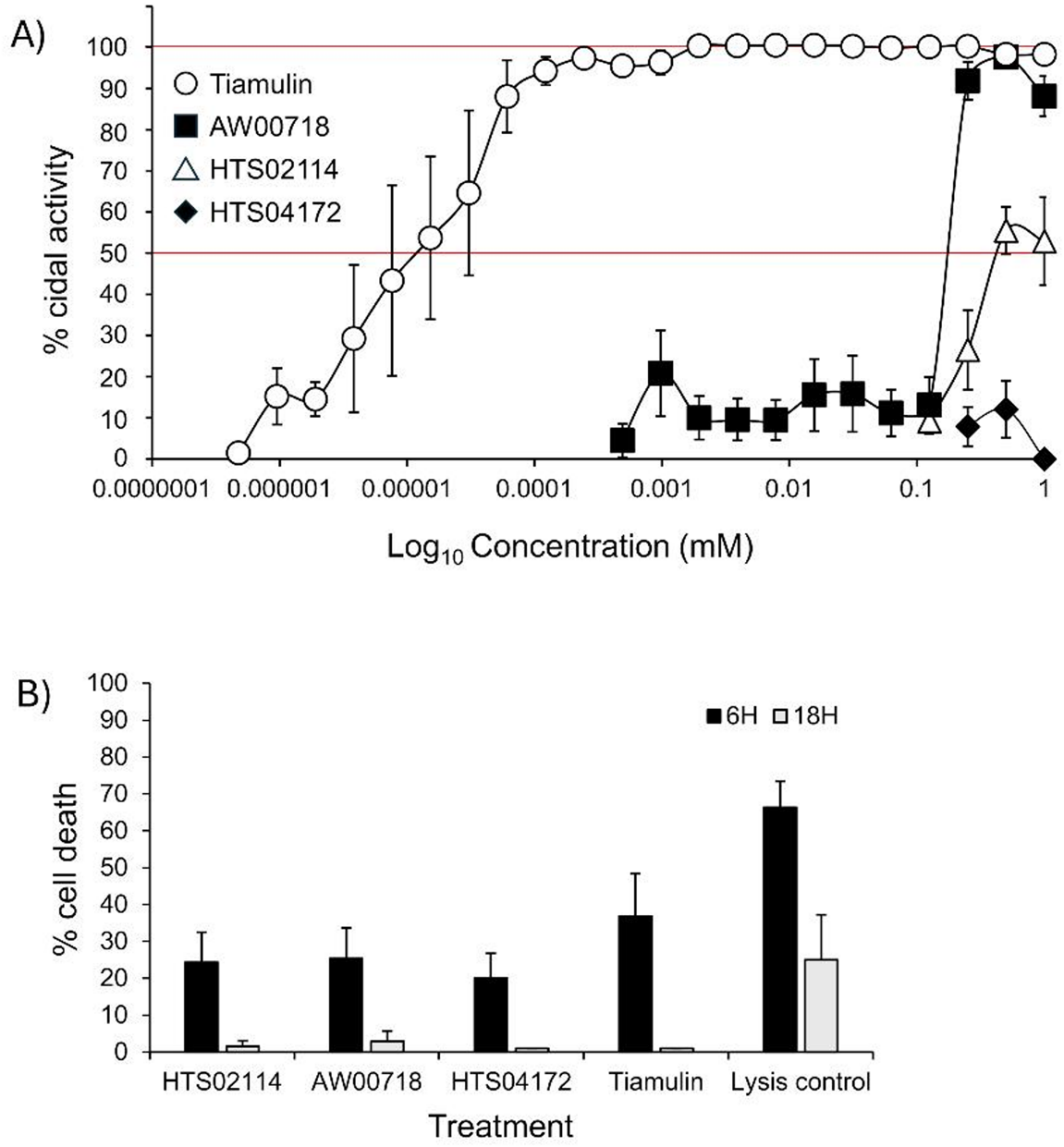
Biological activity of Hit compounds. **(A) Biocidal activity.** Broth microdilution MIC experiments were done with Hit 1, 3 and 4 compounds and the antibiotic tiamulin against a representative *B. pilosicoli* isolate. The symbols represent the mean percentage killing of *n* = 3 (Hit 1 and 4), *n* = 4 (Hit 3) and *n* = 6 (tiamulin) titrations and the error bars the standard errors of the means. **(B) Cytotoxicity**. The cytotoxicity of Hit compounds and tiamulin were assessed on Chang conjunctival epithelial cells in vitro using a resazurin assay. The symbols represent the mean percentage survival of cells after treatment, and the error bars the standard errors of the means of *n* = 6 experiments.

Assessing the bactericidal activity of compounds against *B. pilosicoli* is complicated by (i) the slow and anaerobic growth of the organism, (ii) the absence of Clinical & Laboratory Standards Institute (CLSI) accredited minimum inhibitory and bactericidal concentration (MIC/MBC) protocols for testing new antimicrobials and (iii) the few studies on the mechanisms of antimicrobial action and the development of resistance^19,35^. In general, published studies have used either a broth microdilution assay or an agar dilution method^19,36–39^. In the current study, we assessed the bactericidal activity of the three compounds in vitro using a broth microdilution assay. In developing the assay for *B. pilosicoli*, a 5-day anaerobic incubation was necessary for visible bacterial growth that could be measured by spectrophotometer. This incubation period agreed with the MIC protocol used for testing microbial natural products against *B. hyodysenteriae*^40,41^. Serial dilutions of Hit 1 (compound ID HTS02114), Hit 3 (Compound ID AW00718) and Hit 4 (Compound ID HTS04172) were tested against *B. pilosicoli*, with the antibiotic tiamulin as a control treatment. Hit 4 showed no bactericidal activity against *B. pilosicoli* (MIC > 1mM) and Hit 1 had weak activity with a MIC50 of 0.5 mM (Fig. 11A). Hit 3 was the most effective of the compounds, with values of MIC50 of 0.25 mM and MIC100 of 0.5 mM (Fig. 11A). Tiamulin was highly effective against this representative isolate with MIC50 value of 0.015 µM and MIC100 value of 2µM (Fig. 11A).

The MDS provided insights into the binding interactions and stability of Hit 1, Hit 3, and Hit 4 compounds with Bp-MurI. All three complexes demonstrated stable binding throughout the simulation, albeit with some minor differences between the three Hit compounds in terms of conformational stability, reduced residue fluctuations, and the number of hydrogen bonds formed. These differences may be immaterial to biological efficacy, and the in silico predictions did not match exactly the biological outcomes when the compounds were tested against *B. pilosicoli*, thereby highlighting the importance of experimental testing of new drugs. There are several possible reasons to explain the lack of activity of Hit 4 and the weak activity of Hit 1 compounds. For example, they may be inactivated by components present in the rich growth medium used in the MIC assay, e.g. by adsorption to proteins present in the BHI and FCS. They may also be unable to penetrate the Gram-negative outer membrane (OM) of the organism. Another possible explanation is that they could be subject to active efflux, as putative OM efflux proteins have been identified in the *B. pilosicoli* proteome^42^. By contrast, the most effective Hit 3 compound may not be affected by these processes, although it was still significantly less effective (by a magnitude of 4 log_10_ at the MIC100, and 2 log_10_ for the MIC50) than the currently used antibiotic tiamulin. Moreover, the MM-PBSA results further supported the stability of ligand binding observed during MD simulations and provided a quantitative estimate of binding affinity. Notably, although Hit 4 exhibited the most favourable predicted ΔG_bind, its lack of in vitro antimicrobial activity suggested either poor cellular uptake, poor permeability across the *B. pilosicoli* OM, or rapid efflux. Conversely, Hit 3 demonstrated both favourable free-energy contributions and measurable antimicrobial activity, indicating better alignment between computational binding predictions and biological efficacy.

We also carried out a pilot examination of the cytotoxicity of the compounds using a resazurin assay and found that after treatment with 1mM of each compound for 24 h, cytotoxicity was ~ 20–25% for the Hit compounds and ~ 37% for tiamulin, after 6 h of resazurin exposure (Fig. 11B). After 24 h of resazurin exposure, the cytotoxicity of all compounds fell to zero and to ~ 25% for the antibiotic. The likely explanation for this fall in cytotoxicity is the probable resistance of surviving cells to the single dose of the compounds, with subsequent proliferation. Thus, the compounds are tolerated well by cultured cells, although we appreciate that a limitation of the study was that cytotoxicity was not tested on avian cell lines that are susceptible to *B. pilosicoli* infection. This limitation and the analysis of other markers of cytotoxicity, such as the possible induction of inflammatory and stress responses, could be explored in more detailed future studies to develop a product profile.

We also used the SwissADME platform independently to evaluate their physicochemical properties, pharmacokinetic parameters, and drug-likeness of the Hit compounds compared with tiamulin (Supplementary Fig. 3). The Bioavailability Radar provides a rapid review of drug-likeness, on the evaluated criteria of lipophilicity, size, polarity, solubility, flexibility and saturation^43^. Drugs must fall within the pink areas of the radar to be considered drug-like, and this was generally true for all Hit compounds and tiamulin (Supplementary Fig. 3). For physico-chemistry, the Hit compounds and tiamulin reported moderate solubility according to ESOL, Ali and SILICOS-IT algorithms, with poorly solubility reported only for Hit 1 and Hit 3 by SILICOS-IT and for tiamulin by Ali. However, there were some differences between the hit compounds and tiamulin with respect to pharmokinetics. The Cyp isoenzymes are important for eliminating drugs through metabolic transformation^43^, and inhibition of these isoenzymes might lead to toxicity and accumulation of the drugs or their metabolites. SwissADME identified that Hit 1, 3 and 4 showed interactions with several cytochrome P450 (CYP) inhibitors, whereas tiamulin did not, which suggests unfavorable pharmokinetics for the former. However, for drug-likeness, Hit compounds 1, 3 and 4 and tiamulin complied generally with the Ghose, Veber, Egan, and Muegge pharmaceutical filters and did not violate Lipinski’s Rule of Five. For medicinal chemistry, there were no Pan Assay Interference Compounds (PAINS) alerts detected for any of the molecules. The Brenk parameter provides a measure of putative toxicity, chemical reactivity and metabolic instability that would lead to poor pharmacokinetics; there were no alerts for Hit 1, 3 or 4 compounds, but surprisingly a violation for tiamulin with the presence of an isolated alkene. Finally, the medicinal chemistry section provides a measure of ‘lead-likeness’, which defines a Hit compound as one that is favorable for optimisation; all three Hit compounds showed two violations of this parameter, with MW > 350 and SLOGP3 > 3.5, which was less than tiamulin, which showed three violations of MW > 350, Rotors > 7 and XLOGP3 > 3.5. Thus, SwissADME provided a rapid prediction of key parameters for the Hit compounds, which overall compared favorably with tiamulin.

### Diversity of the glutamate racemase in B. pilosicoli isolates

Hit 3 compound was the most effective of the compounds identified in silico against *B. pilosicoli*. One consideration for development of Hit-to-lead is whether the binding site of the enzyme targeted by Hit 3 is conserved, and thus the active compound can bind to *Mur*I of different isolates. To examine this, we accessed the available *B. pilosicoli* genomes from Genbank and downloaded the amino acids sequences for the glutamate racemase from 87 entries. Of these, eight amino acid sequences were partial and were excluded, one isolate did not contain the protein, and of the remainder, 16, 12, 4 and 2 isolates respectively, shared the same identifier and thus 15, 11, 3 and 1 additional identical sequences were excluded. Thus, the remaining 48 unique sequences were aligned with Clustal (Supplementary Fig. 4): overall, there was ~ 71% amino acid identity across the whole protein (1-267 amino acids) and 79% identity across amino acids 1-239. When the critical amino acid residues involved in the interaction with Hit 3 were examined, the majority of the amino acid residues implicated in Chain A and B (Table 2) were conserved across the 48 isolates, with only three isolates having Asp replaced by Glu at position 211 of Chain A and two isolates each having Thr replaced at position 214 of Chain A with either Asn or Gln (Supplementary Fig. 4). Thus, it appeared that Hit 3 compound would be able to interact with MurI from 85% of the isolates examined.

## Conclusions

In summary, in this pilot study, we used a computational approach with HTVS to identify potential small molecule inhibitors targeting the MurI (glutamate racemase) enzyme of *B. pilosicoli*, one of which showed bactericidal activity in vitro. The pilot study provided proof-of-concept of the in silico approach for finding new antimicrobial compounds against the pathogenic *Brachyspira*: it does, however, have some limitations. First, the Maybridge library has ~ 51,000 compounds, and interrogating larger chemical compounds, e.g. MCULE^44^ and natural product libraries could provide more Hit compounds for testing. Furthermore, it is possible that the other Hit compounds identified in Table 1 could have biological activity against *B. pilosicoli* and may deserve testing regardless of their in silico predictions. However, the Hit 1, Hit 3 and Hit 4 structures identified thus far could themselves serve as templates for analogue chemistry to develop more bioactive compounds. Secondly, experimental confirmation of the binding of protein and inhibitors was not attempted, which would require the production of recombinant Bp-MurI and biophysical measurements such as enzyme inhibition assays, thermal shift analysis, and co-crystallisation studies^45^. Such approaches would depend on the ability to produce Bp-MurI in its native conformation, which could be difficult if the expressed recombinant protein was insoluble. Thirdly, examination of the mode of action of these compounds is complicated by the slow and anaerobic growth of the organism. Tiamulin is principally a semi-synthetic bacteriostatic antibiotic that obstructs the 50 S ribosomal subunit within bacteria and inhibits the peptidyl transferase enzyme^46^, and it is also bactericidal when used in high concentrations^47^. By contrast, the mechanism of action of Hit 3 compound is not known, beyond its predicted in silico binding to Bp-MurI. D-glutamate is an essential building block for peptidoglycan, and it is produced from L-glutamate by the MurI enzyme, and perturbation in MurI enzyme activity would be predicted to have downstream effects on the cell wall. Although the identified compounds were designed to target the MurI active site and demonstrated stable binding in silico, we acknowledge that direct experimental validation of MurI inhibition was not performed in this pilot study. Therefore, target engagement remains inferred and represents an important focus for future biochemical and structural investigations.

Small molecule inhibitors to the MurI enzymes of other bacteria have been reported in a few studies. For example, Lundqvist et al. used a high-throughput screen against the AstraZeneca compound collection (385,861 compounds) and identified a pyrazolopyrimidinedione analogue that specifically bound to a cryptic allosteric site of the *H. pylori* MurI^45^: however, no microbicidal data were included. Pawar et al. used a computational approach to screen various classes of natural compounds for their binding to the *Mycobacterium tuberculosis* MurI^22^. The best binding was exhibited by the flavonoids naringenin and quercetin, which inhibited racemization activity with induced structural perturbation of *M. tuberculosis* MurI. When tested against fast-growing *M. smegmatis* bacteria, both flavonoids inhibited bacterial growth, which was accompanied by membrane damage and morphological changes^22^. More recently, HTVS and MDS identified novel potential compounds with good fitness and docking scores for the MurI enzyme of *Neisseria gonorrhoeae*, but no experimental validation was done^26^. Hypothetically, the interactions of Hit 3 compound and the Bp-MurI could lead to perturbations in the cell wall via disrupted peptidoglycan synthesis, which eventually leads to bacterial death. Future studies would test this hypothesis and examine whether induced changes or the minor natural variation in the amino acids of the MurI binding sites for Hit 3 compound, would ameliorate the bactericidal effect.

On a final note, recent advances in artificial intelligence (AI) and machine learning (ML) models are enhancing the efficiency and predictive power of structure-based drug discovery pipelines, particularly in high-throughput virtual screening (HTVS). AI-driven approaches have been successfully applied to diverse therapeutic targets, enabling improved ligand prioritisation, binding pose refinement, and identification of novel druggable sites. For instance, integrative computational strategies combining virtual screening with dynamic simulations have facilitated the identification of potential antiviral targets and ligands in emerging pathogens such as monkeypox virus^48^. Similarly, pharmacophore-based screening frameworks, when coupled with advanced computational optimisation, have been effectively used to identify enzyme inhibitors in complex diseases such as Alzheimer’s disease^49^ and cancer^50^. These developments highlight the growing convergence of AI-assisted modelling with traditional molecular docking and simulation approaches, improving the accuracy and translational relevance of in silico predictions. In this context, our study employs a structured computational pipeline integrating pharmacophore modelling, molecular docking, and molecular dynamics simulations to identify potential inhibitors of Bp-MurI, reflecting the broader paradigm shift towards data-driven and computationally augmented drug discovery.

## Methods

### Protein modelling

The amino acid sequence of Bp-MurI, comprising 267 residues, was retrieved from the National Center for Biotechnology Information (NCBI) with Uniprot accession no. AGA65885.1. To obtain a reliable three-dimensional (3D) structure, we used a template-based protein modelling approach. The structural template was identified through Protein Data Bank (PDB) structural BLAST, multiple sequence alignment, and homology assessment. Considering high degrees of query coverage and homology, the crystal structure of *Staphylococcus aureus* glutamate racemase in complex with D-Glutamate (PDB ID: 2JFQ) was selected as the template. AlphaFold^51^ was used for protein modelling to generate a robust and accurate 3D representation of Bp-MurI for subsequent analyses.

### Protein model validation

To ensure the accuracy and reliability of the Bp-MurI model, the model was first superimposed onto its respective template structure (2JFQ) using the PyMol molecular visualisation system (version 2.4.0)^52^. The root mean square deviation (RMSD) of coordinates between the homology model and the template structure was calculated, providing a quantitative measure of structural similarity. SWISS-MODEL^53^, Phyre2^54^, and Bhageerath-H^55^ were used for independent validation and comparative analysis of our predicted/generated model. These servers employ diverse algorithms and methodologies, thereby enhancing the robustness of the validation process.

The accuracy and stereo-chemical properties of the predicted model were assessed using the PROCHECK server^56^. The Ramachandran plot and overall goodness factor (G-factor) were used to evaluate the model’s conformational quality. The model’s accuracy was scrutinised through the PDBsum online server^57^, providing a fine assessment of its structural integrity. The overall quality and reliability of the Bp-MurI homology model were further evaluated using the ERRAT program, which analyses the statistics of non-bonded atom-atom interactions to detect regions of probable error in the model. ERRAT was accessed through the SAVES v6.0 web server (https://saves.mbi.ucla.edu/), providing an overall quality factor where higher scores indicate better model quality^58^. These validation steps collectively ensured the fidelity of the Bp-MurI model, establishing a reliable step for further analyses.

### Identification of binding site

DeepSite^59^ was used to determine the active site crucial for ligand binding within the Bp-MurI structure. This method integrates advanced deep learning algorithms, enhancing the accuracy of identifying potential ligand-binding regions. To strengthen the reliability of the predicted binding site, we applied a consensus approach, cross-referencing the outcomes with other contemporary tools for binding site prediction. This multi-faceted analysis ensured an in-depth exploration of potential ligand interaction sites, optimising the accuracy of subsequent computational analyses. The consensus prediction helps to develop a robust understanding of the Bp-MurI binding site, laying the groundwork for virtual screening and molecular docking analyses. This strategic approach avoids redundancy while facilitating a thorough exploration and validation of potential ligand interaction sites.

### Structure-based pharmacophore generation

To generate the structure-based pharmacophore, Bp-MurI was selected as the receptor and D-Glutamate as the ligand. Docking of the substrate D-Glutamate into the conserved binding site of the modelled Bp-MurI receptor, as predicted in the preceding step, facilitated the selection of the optimal docking pose. Criteria for selection included the highest docking score and the lowest root mean square deviation (RMSD) concerning D-Glutamate in the crystal structure of *Staphylococcus aureus* glutamate racemase. The structure-based pharmacophore model was then generated with the ‘receptor-ligand pharmacophore generation module’ of Biovia DS 2020. This software deciphers ligand-receptor interactions based on essential pharmacophore features, e.g. hydrogen bond donor and acceptor, and hydrophobic and hydrophilic regions. To preserve the steric integrity of the macromolecule and identify an optimal pharmacophore, an ‘excluded volume’ was incorporated into the active site, as recommended in previous studies^60^.

### Database preparation, high-throughput virtual screening

A library of drug-like compounds containing approximately 51,000 compounds from the Maybridge database (Supplementary Dataset 1) was used and mapped onto the generated pharmacophore model using the Biovia DS 2020 screen library module. Following the primary round of screening, which was based on pharmacophore mapping and fit value cut-off, the compounds were further filtered using ADMET (Absorption, Distribution, Metabolism, Excretion And Toxicity), TOPKAT (TOxicity Prediction by Komputer Assisted Technology), VEBER^61^, and Lipinski’s filter^62^. Virtual screening is essential for in silico drug discovery process as it hastens drug development^63^ and selection of the best candidate drugs. A pharmacophore-based approach was needed for the most energetically favourable site that discovers matching pharmacophores in the ligands, and a minimum of 4–5 sites are required to match hypotheses with 8–10 sites for filtering the database molecules. This criterion was followed in the present work to screen the Maybridge database. Also, The Maybridge HitFinder library was selected because it represents a highly curated collection of ~ 51,000 structurally diverse, drug-like small molecules specifically assembled to maximize scaffold novelty. Unlike larger commercial libraries, HitFinder contains only purity-verified compounds with favourable physicochemical profiles, enabling rapid downstream procurement and experimental validation. Its extensive prior use in antibacterial lead discovery—particularly for targets lacking known inhibitors—makes it an appropriate choice for initial hit identification against Bp-MurI. Database hits were initially ranked according to their fitness scores, evaluating how closely each aligned ligand conformer matched the hypothesis based on RMSD and site matching^64^. Molecules identified through High-Throughput Virtual Screening (HTVS) were then subjected to molecular docking. Prior to docking, all hit compounds underwent energy minimization using the steepest descent method for 3,000 steps, followed by the conjugate gradient method for 3,000 steps, utilizing the Biovia DS 2020 energy minimization tool^65^. Molecular docking was performed using the Biovia DS 2020 CDOCKER module, and compounds exhibiting the highest docking scores, considering both CDOCKER energy and fit value, were shortlisted.

### Molecular docking (MD) studies

From HTVS, three top Hit compounds were shortlisted as novel compounds and were used for further MD studies. The novel small molecule inhibitor compounds were characterised, and energy minimised using conjugate gradient and steepest descent methods and docked with Bp-MurI using Biovia Discovery Studio 2020^66–69^, an open-source program for MD. The docking scores were obtained in terms of a combined scoring function, which calculates the affinity, or fitness, of protein-ligand binding by summing up the contributions of several individual terms.

To ensure the reliability of the docking protocol, the natural substrate D-glutamate was docked into the predicted active site, and the resulting pose was evaluated based on docking score and structural alignment with the template-bound conformation. This substrate-guided validation step ensured accurate reproduction of biologically relevant interactions within the catalytic pocket.

### Multifaceted analysis of crucial protein-ligand interactions

A comprehensive analysis was done using Biovia Discovery Studio 2020 to understand the crucial molecular interactions governing the binding affinity between Bp-MurI and the identified Hit compounds (Hit 1, Hit 3, and Hit 4). This methodology encompasses the evaluation of two-dimensional (2D) and 3D interaction diagrams along with hydrophobicity, to shed light on details of the binding interfaces. Interaction diagrams in 2D were generated to provide a visual representation of the specific interactions between each Hit compound and Bp-MurI. Key interactions, e.g. hydrogen bonding, hydrophobic interactions, and pi-stacking, were carefully annotated for a detailed understanding. Interaction diagrams in 3D were also constructed to offer spatial perspective on the binding configurations. The 3D representation aids in visualising the orientation and positioning of Hit compounds within the active site of Bp-MurI.

The hydrophobicity profile of the binding pocket was assessed to discern the nature of the interactions between Bp-MurI and the lead compounds. The hydrophobicity scale was categorised into highly negative, intermediate negative, around zero, intermediate positive, and highly positive values, each indicative of distinct characteristics in amino acid behaviour.

1. Highly Negative Values (Close to -3.00): Amino acids in this range exhibit high hydrophilicity, often interacting strongly with water molecules. These residues are typically found on the protein surface, contributing to solubility, and forming hydrogen bonds with water.
2. Intermediate Negative and Positive Values: Moderately hydrophilic and hydrophobic amino acids were observed, showcasing varied behaviour based on their location within the protein structure.
3. Values Around Zero: Amino acids in this range display balanced behaviour, interacting with both water and non-polar substances. Their location within protein structures is context dependent.
4. Highly Positive Values (Close to 3.00): Amino acids with high hydrophobicity avoid water interaction and are crucial for forming a stable hydrophobic core in the protein interior.

### Molecular dynamics simulations (MDS)

MDS were done to assess the stability and dynamic properties of the Bp-MurI model when bound to the newly identified novel small molecule inhibitors. The GROMACS 2020.1 package^70^ subjected the Bp-MurI model complexed with the novel inhibitors to an extensive 100 ns MD simulation. The force fields for the protein were generated using the Gromos96 43a1 force field^70^, while the ligand topology force fields for both reference and potential inhibitors were crafted using the Prodrg server^71^. The systems were solvated in a cubic box of extended simple point-charge (SPC) water molecules, and charge neutrality was achieved through the strategic addition of Na^+^ and Cl^−^ ions. Energy minimisation, using the steepest descent method for 5000 steps, was undertaken to alleviate short-range unfavourable contacts. To ensure full reproducibility, we report additional system-level details for all simulations. Each protein–ligand complex consisted of approximately 75,000–80,000 atoms within a cubic box measuring ~ 9.5 × 9.5 × 9.5 nm³, solvated using SPC/TIP3P-equivalent water and neutralized with the appropriate number of Na⁺ and Cl⁻ ions. Energy minimization was performed using 5000 steps of the steepest descent algorithm with a force tolerance of 1000 kJ/mol/nm. This was followed by 500 ps of NVT and 1 ns of NPT equilibration using the V-rescale thermostat (298 K) and Parrinello–Rahman barostat (1 bar). Production simulations were run for 100 ns with a 2 fs timestep under PME electrostatics (real-space cut-off 1.0 nm), LINCS constraints, and a 1.0 nm Lennard–Jones cut-off.

Starting with position-restrained simulations at 298 K for 100 ps, the three complexes underwent a 100 ns MD production run, alongside a control run for the reference compound (Bp-MurI unbound). Throughout the simulations, temperature (298 K) and pressure (1 bar) were maintained using the V-rescale thermostat and the Parrinello–Rahman method^72^, respectively. The Particle Mesh Ewald (PME) method^73^ handled long-range electrostatic forces, ensuring precision with a real-space cut-off of 10 Å, PME order of six, and a relative tolerance between long and short-range energies of 10 − 6 kcal/mol. Short-range interactions were evaluated through a neighbour list of 10 Å updated every 10 steps. Lennard-Jones (LJ) and real-space electrostatic interactions were truncated at 9 Å. Hydrogen bond constraints were applied using the LINCS algorithm^74^.

Various analyses were done to assess stability, including potential energy, Root Mean Square Deviation (RMSD), Root Mean Square Fluctuations (RMSF), Solvent-Accessible Surface Area (SASA), hydrogen bond dynamics, and the radius of gyration (RoG)^75^. Conformational variations in the Bp-MurI-substrate and Bp-MurI-inhibitor complexes were elucidated by calculating RMSD values for Ca atoms, providing a deeper understanding of the dynamic behaviour. The final models were derived by averaging snapshots from the MD trajectory post-stabilisation, culminating in a broad assessment of the structural subtlety and stability of the studied complexes.

### Molecular mechanics Poisson–Boltzmann surface area (MM-PBSA) calculations

To further refine the binding energetics obtained from molecular docking and MD simulations, Molecular Mechanics Poisson–Boltzmann Surface Area (MM-PBSA) calculations were performed using the gmx_MMPBSA package (v1.x), which is compatible with GROMACS output trajectories. The final 20 ns of each 100-ns MD trajectory were extracted, and frames were sampled every 100 ps, yielding 200 snapshots per system. These snapshots were used for ensemble-averaged free-energy estimation.

The binding free energy (ΔG_bind) was computed according to:

ΔGbind = ΔEvdW + ΔEelec + ΔGsolvpolar + ΔGsolvnonpolar\Delta G_{bind} = \Delta E_{vdW} + \Delta E_{elec} + \Delta G_{solv}^{polar} + \Delta G_{solv}^{nonpolar}ΔGbind​=ΔEvdW​+ΔEelec​+ΔGsolvpolar​+ΔGsolvnonpolar​.

where.

- ΔE_vdw and ΔE_elec represented van der Waals and Coulombic interactions,
- ΔG_solv^polar was computed using the Poisson–Boltzmann (PB) model with a protein dielectric constant of ε = 2 and solvent dielectric of ε = 80,
- ΔG_solv^nonpolar was estimated using a solvent accessible surface area (SASA) model with γ = 0.0227 kJ/mol/Å².

All calculations were done using the single-trajectory approach, ensuring consistent structural alignment of protein and complexes. Energy decompositions were analyzed to identify the dominant contributors to binding.

### Bactericidal activity of compounds against B. pilosicoli in vitro

A broth microdilution assay was established to determine the minimum inhibitory concentrations (MIC) of the Hit compounds 1, 3 and 4 against *B. pilosocoli* strain BP2904^76^. The bacterium was grown from − 80 °C glycerol frozen stocks for 5 days on fastidious anaerobe agar (LAB090), supplemented with defibrinated sheep blood (5 % (v/v), E&O Laboratories, Bonnybridge, Scotland) at 37 °C in an anaerobic gas jar with the Anaerogen gas-generating system (Oxoid, UK). The Hit compounds from Maybridge (ThermoScientific, UK) and the antibiotic tiamulin fumarate (Merck, UK, cat. no. 46959) were dissolved in dimethyl sulphoxide (DMSO, 100%) and tested in a two-fold dilution series in triplicate starting from 1mM final concentration/well. The wells contained 180 µL of a bacterial suspension of 5 × 10^5^ colony forming units/mL in Brain Heart Infusion Broth supplemented with 10% (v/v) decomplemented foetal calf serum (Lonza, United Kingdom), to which was added 20 µL of the serial dilutions of the test compound(s). Negative control was medium alone. No treatment control contained 180 µL of a bacterial suspension of 5 × 10^5^ colony forming units/mL made up to a final volume of 200 µL with 20 µL of medium. The plates were incubated under anaerobic conditions as described above and a period of 5 days was required to get sufficient bacterial growth that could be measured in a spectrophotometer (SpectraMax iD3, Molecular Devices, San Jose, CA, USA) at λ600nm. The percentage bactericidal activity of the compounds and antibiotic were calculated from the equation = 100-(Absorbance of test/Absorbance of control)) x 100. The MIC50 and MIC > 90 values were defined as the concentrations that gave 50% and > 90% reduction in absorbance values compared to the control. DMSO alone had no bactericidal effect on *B. pilosicoli.*

### Cytotoxicity of hit compounds

Cytotoxicity was assessed as described recently^77^. Briefly, Human Chang conjunctival epithelial cells (European Type Culture Collection, Porton Down, United Kingdom) were cultured in the wells of sterile 96-well cell culture plates (Nunc) at 37 °C with 5% (v/v) CO_2_ in Dulbecco’s modified Eagle’s medium supplemented with Glutamax-1 and sodium pyruvate (DMEM) (Lonza, United Kingdom) and 10% (v/v) decomplemented fetal calf serum (dFCS) (Lonza, United Kingdom). Prior to treatment, the medium was removed, fresh medium added (180 µL/well) and then 20 µL of Hit compounds were added per well. Lysis solution (1% (w/v) sodium dodecyl sulfate in 0.1 M NaOH) was used as a positive control, and negative controls contained cells alone (no treatment). The plates were incubated for 18 h at 37 °C with 5% (v/v) CO_2_, and then 20 µL of resazurin (Merck, United Kingdom) was added to each well. The plate was incubated for a further 6 and 18 h, and the absorbance was read at λ = 570 and λ = 595 nm for background correction (SpectraMax iD3 plate reader). Cytotoxicity was calculated as described previously^78^.

The SwissADME platform (http://www.swissadme.ch/) was used to assess physicochemical properties, pharmacokinetic behavior, drug-likeness, and medicinal chemistry parameters^43^. This in silico tool evaluated physicochemical characteristics such as the number of rotatable bonds, hydrogen bond donors and acceptors; Absorption, Distribution, Metabolism, Excretion, and Toxicity (ADMET) properties; expressed as log P lipophilicity; log S hydrophilicity; pharmacokinetic parameters, e.g. predicted gastrointestinal absorption and cytochrome P450 inhibition profiles, etc.; drug-likeness based on major pharmaceutical industry filters of Lipinski, Veber, Egan, and Muegge; and Pan-Assay Interference Compounds (PAINS) alerts.

## Supplementary Information

Below is the link to the electronic supplementary material.

Supplementary Material 1

Supplementary Material 2

## Data Availability

All computational modelling data are presented within the manuscript and supplementary materials. The raw data for the MIC and cytotoxicity experiments generated during the current study are available from the corresponding author, if required, on reasonable request.
