# Supplementary material for: Identification of small molecule inhibitors for the Brachyspira pilosicoli glutamate racemase (Bp-MurI) enzyme using a computational and experimental approach
Source: Sci Rep. 2026 Apr 2;16:15632. doi: 10.1038/s41598-026-46506-w (PMC13186945; doi:10.1038/s41598-026-46506-w)
Supplement: Supplementary file 2 — Supplementary Material 2 [file 41598_2026_46506_MOESM2_ESM.pdf]

Identification of small molecule inhibitors for the *Brachyspira pilosicoli* glutamate racemase (Bp-MurI) enzyme using a computational and experimental approach

Authors: Ravi Kant[1,2], Roberto La Ragione[3,4], Myron Christodoulides[1*]

Affiliations

[1] Molecular Microbiology, School of Clinical and Experimental Sciences, Faculty of Medicine, University of Southampton, Southampton, England, SO16 6YD.

[2] Faculty of Applied Sciences & Biotechnology, Shoolini University, Solan, Himachal Pradesh - 173229, India.

[3] School of Biosciences, Faculty of Health and Medical Sciences, Edward Jenner Building, University of Surrey, Guildford, England, GU2 7XH.

[4] School of Veterinary Medicine, Faculty of Health and Medical Sciences, University of Surrey, Guildford, England, GU2 7AL.

***Author for correspondence:** Molecular Microbiology, School of Clinical and Experimental Sciences, Faculty of Medicine, University of Southampton, Southampton, England, SO16 6YD.

E-mail: mc4@soton.ac.uk

SUPPLEMENTARY INFORMATION

1. Estimation of annual global losses to the porcine and poultry industries from PIS and AIS (calculation generated by AI tool).

There are no robust, peer-reviewed global totals available for either disease, so any “global loss” is necessarily a coarse estimate built from local studies and scaling assumptions. Below (A) what published numbers exist, (B) a transparent scaling method and math, and (C) a defensible range for annual global losses with citations.

A. What the literature says (key facts)

1) **PIS = porcine intestinal (or colonic) spirochaetosis**, caused mainly by *Brachyspira pilosicoli*. It produces diarrhoea, reduced average daily gain (ADG) and worse feed conversion in growing pigs; studies note measurable productivity losses, but no published global cost estimate was found [1,2].
2) **AIS = avian intestinal spirochaetosis**, caused by *Brachyspira* spp. in laying hens; several UK studies/estimates put the UK annual cost at ≈£14–18 million/year. There are published estimates of significant regional losses but few global aggregated figures [3-5].
3) For context, the global poultry market (meat + eggs) is on the order of ~USD 325–375 billion / year (market reports) and the global egg/laying-hen population is ~~7.9 billion layers (FAO / industry summaries). The global pork market is in the ~USD 290–400+ billion / year range and global pig numbers are reported in FAO/OurWorldInData datasets (hundreds of millions to ~1.5 billion animals depending on counting snapshot vs annual throughput). The Business Research CompanyCompassion in World FarmingPrecedence ResearchOur World in Data

B. Estimation approach (transparent, reproducible)

Because only national estimates (UK) for AIS and farm/experimental impacts for PIS were located, a simple scaling method was used.

AIS — scaling from UK layer estimate

1) Source: UK AIS loss estimate ≈ £14–18M/year (based on a UK laying flock ~30 million hens in older studies).
2) Global number of layers ≈ 7.9 billion (FAO summaries). Compassion in World Farming
3) Scale factor = 7.9B / 30M ≈ 263.3.
4) Scaled global AIS loss ≈ £14M × 263 → £3.7 billion up to £18M × 263 → £4.7 billion per year.
5) Convert to USD (approx): using a rough GBP→USD rate ~1.27 (rate varies) gives ~USD 4.7–6.0 billion / year.
(This assumes UK per-bird impact is representative globally, which is a big assumption; see uncertainty below.)

PIS — no published global cost; construct scenario ranges

1) There is no clear global monetary estimate in the literature for PIS. The disease is documented to reduce ADG and feed conversion and thus raise production cost, but reported effect sizes and herd prevalence vary by region and production system.
2) To produce a useful *order-of-magnitude* range, a scenario logic is applied to the global pork market value (≈USD 290–400B): if PIS (through reduced growth/feed efficiency and occasional mortality) causes a net productivity/market-value loss of:

- 0.5% → loss ≈ USD 1.5–2.0 billion/year
- 1.0% → loss ≈ USD 2.9–4.0 billion/year
- 2.0% → loss ≈ USD 5.8–8.0 billion/year

3) These percentages are plausible given documented reductions in ADG and feed conversion with enteric *Brachyspira* infections, but they are illustrative — the real figure depends on prevalence of clinical disease (which varies widely), production system, and mitigation (treatment, biosecurity) [6] The Pig Site

C. Final numbers (best-effort ranges, with uncertainty)

1) AIS (poultry, global) — ≈ £3.7–4.7 billion / year (≈ USD 4.7–6.0 billion / year) using UK per-layer losses scaled to global layer numbers. Uncertainty: high (could be substantially lower or higher depending on regional prevalence, egg system type, antibiotic usage, detection improvements).
2) PIS (porcine, global) — no direct published global total. Using plausible productivity-loss scenarios applied to the global pork market (≈USD 290–400B) gives a very rough range of ~USD 1.5 billion (0.5%) up to ~USD 8+ billion (2%) per year. USD 2–6 billion/year is a plausible central band given current evidence, but stress this is a scenario-based estimate rather than an empirical global accounting. Precedence ResearchPMC

D. Main caveats & where the big uncertainties come from
1) Prevalence & detection: AIS/PIS may be under-detected in many countries; UK estimates were made when layer numbers and antibiotic practices differed from today. Scaling implicitly assumes similar prevalence and impact per bird/pig worldwide, which is unlikely.
2) Impact heterogeneity: Effects differ by production system (intensive vs backyard), region (feed types, co-infections), and control measures (antibiotics, vaccination where applicable). A 1% productivity hit in a high-value market is much more costly (in $) than in a low-input system. The Pig Site
3) Time variability: Recent severe events (HPAI for poultry, ASF/PRRS for pigs) can dwarf enteric disease impacts in some years and regions; those are separate diseases but influence market baselines. The Guardian Iowa State Research

References

1. Hampson DJ (2018). The Spirochete *Brachyspira pilosicoli*, Enteric Pathogen of Animals and Humans. Clin Microbiol Rev 31(1)
2. CABI. Porcine intestinal spirochaetosis. Wallingford, UK, 2019
3. Burch DG, Harding C, Alvarez R, Valks M (2006). Treatment of a field case of avian intestinal spirochaetosis caused by Brachyspira pilosicoli with tiamulin. Avian Pathol 35(3):211
4. Le Roy CI, Mappley LJ, La Ragione RM, Woodward MJ, Claus SP (2015). *Brachyspira pilosicoli*-induced avian intestinal spirochaetosis. Microb Ecol Health Dis 26:28853
5. El-Ghany WAA (2025). Avian intestinal spirochaetosis: an emerging zoonosis. Animal Diseases 5(1):22
6. Smith JL (2005). Colonic spirochetosis in animals and humans. J Food Prot 68(7):1525

Figure S1: Ramachandran Plot generated from PDBsum server to validate the modeled protein structure.

PROCHECK summary for 2jfq

Ramachandran plot

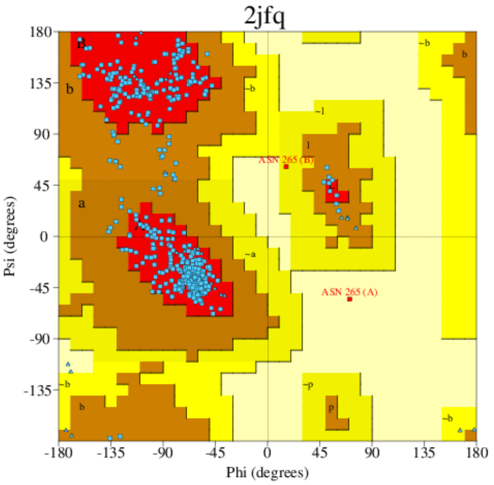

PROCHECK statistics

1. Ramachandran Plot statistics

		No. of residues	%-tage
Most favoured regions	[A,B,L]	423	91.2%
Additional allowed regions	[a,b,l,p]	39	8.4%
Generously allowed regions	[~a,~b,~l,~p]	1	0.2%
Disallowed regions	[XX]	1	0.2%*
Non-glycine and non-proline residues		464	100.0%
End-residues (excl. Gly and Pro)		4	
Glycine residues		38	
Proline residues		26	
Total number of residues		532	

Based on an analysis of **118** structures of resolution of at least **2.0** Angstroms and *R*-factor no greater than **20.0** a good quality model would be expected to have over **90%** in the most favoured regions [A,B,L].

Figure S2: ERRAT analysis using the SAVES v6.0 server shows the overall quality factor of the modeled protein structure.

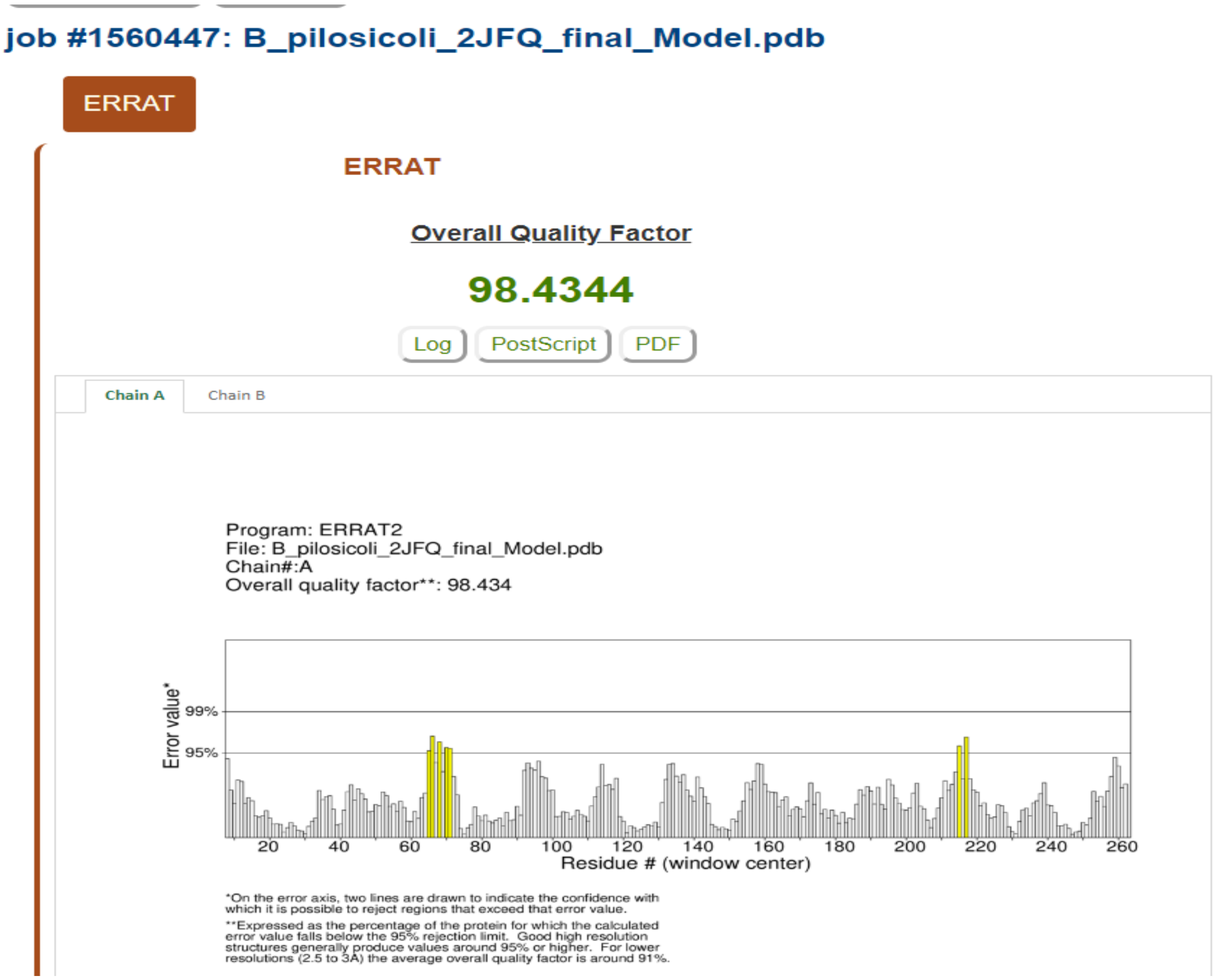

Figure S3. ADMET analysis of Hit compounds and tiamulin

Hit compound 1

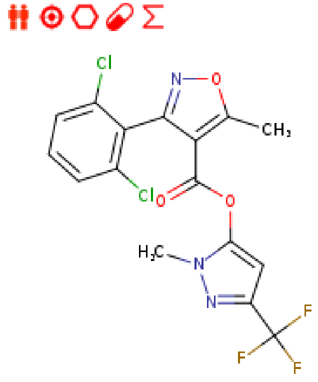

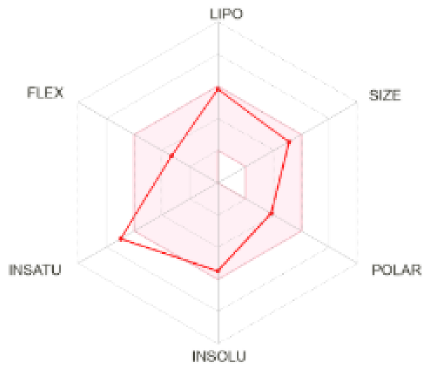

SMILES O=C(c1c(C)onc1c1c(Cl)cccc1Cl)Oc1cc(nn1C)C(F)(F)F

Physicochemical Properties	
Formula	C16H10Cl2F3N3O3
Molecular weight	420.17 g/mol
Num. heavy atoms	27
Num. arom. heavy atoms	16
Fraction Csp3	0.19
Num. rotatable bonds	5
Num. H-bond acceptors	8
Num. H-bond donors	0
Molar Refractivity	90.37
TPSA	70.15 Å²

Lipophilicity	
Log $P_{o/w}$ (iLOGP)	3.20
Log $P_{o/w}$ (XLOGP3)	4.66
Log $P_{o/w}$ (WLOGP)	6.08
Log $P_{o/w}$ (MLOGP)	3.93
Log $P_{o/w}$ (SILICOS-IT)	4.63
Consensus Log $P_{o/w}$	4.50

Water Solubility	
Log *S* (ESOL)	-5.49
Solubility	1.36e-03 mg/ml ; 3.24e-06 mol/l
Class	Moderately soluble
Log *S* (Ali)	-5.86
Solubility	5.80e-04 mg/ml ; 1.38e-06 mol/l
Class	Moderately soluble
Log *S* (SILICOS-IT)	-6.77
Solubility	7.10e-05 mg/ml ; 1.69e-07 mol/l
Class	Poorly soluble

Pharmacokinetics	
GI absorption	High
BBB permeant	No
P-gp substrate	No
CYP1A2 inhibitor	Yes
CYP2C19 inhibitor	Yes
CYP2C9 inhibitor	Yes
CYP2D6 inhibitor	No
CYP3A4 inhibitor	No
Log K_p (skin permeation)	-5.55 cm/s

Druglikeness	
Lipinski	Yes; 0 violation
Ghose	No; 1 violation: WLOGP>5.6
Veber	Yes
Egan	No; 1 violation: WLOGP>5.88
Muegge	Yes
Bioavailability Score	0.55

Medicinal Chemistry	
PAINS	0 alert
Brenk	0 alert
Leadlikeness	No; 2 violations: MW>350, XLOGP3>3.5
Synthetic accessibility	3.37

Hit compound 3

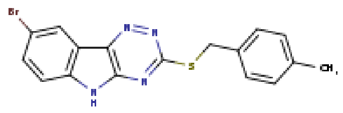

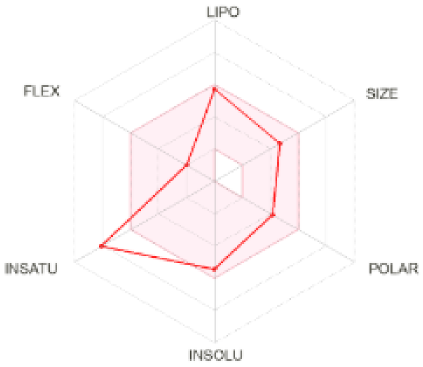

SMILES Cc1ccc(cc1)CSc1nnc2c(n1)[nH]c1c2cc(cc1)Br

Physicochemical Properties	
Formula	C17H13BrN4S
Molecular weight	385.28 g/mol
Num. heavy atoms	23
Num. arom. heavy atoms	19
Fraction Csp3	0.12
Num. rotatable bonds	3
Num. H-bond acceptors	3
Num. H-bond donors	1
Molar Refractivity	98.06
TPSA	79.76 Å²

Lipophilicity	
Log $P_{o/w}$ (iLOGP)	3.27
Log $P_{o/w}$ (XLOGP3)	4.48
Log $P_{o/w}$ (WLOGP)	4.72
Log $P_{o/w}$ (MLOGP)	4.01
Log $P_{o/w}$ (SILICOS-IT)	5.04
Consensus Log $P_{o/w}$	4.30

Water Solubility	
Log *S* (ESOL)	-5.46
Solubility	1.32e-03 mg/ml ; 3.43e-06 mol/l
Class	Moderately soluble
Log *S* (Ali)	-5.88
Solubility	5.14e-04 mg/ml ; 1.33e-06 mol/l
Class	Moderately soluble
Log *S* (SILICOS-IT)	-8.05
Solubility	3.45e-06 mg/ml ; 8.95e-09 mol/l
Class	Poorly soluble

Pharmacokinetics	
GI absorption	High
BBB permeant	No
P-gp substrate	Yes
CYP1A2 inhibitor	Yes
CYP2C19 inhibitor	Yes
CYP2C9 inhibitor	Yes
CYP2D6 inhibitor	Yes
CYP3A4 inhibitor	Yes
Log K_p (skin permeation)	-5.47 cm/s

Druglikeness	
Lipinski	Yes; 0 violation
Ghose	Yes
Veber	Yes
Egan	Yes
Muegge	Yes
Bioavailability Score	0.55

Medicinal Chemistry	
PAINS	0 alert
Brenk	0 alert
Leadlikeness	No; 2 violations: MW>350, XLOGP3>3.5
Synthetic accessibility	2.72

Hit compound 4

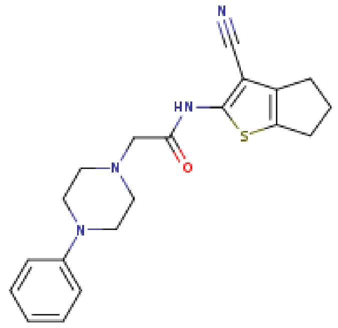

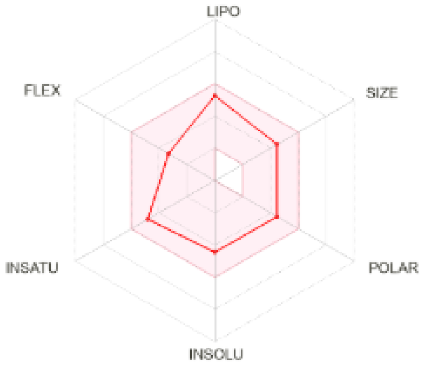

SMILES N#Cc1c(NC(=O)CN2CCN(CC2)c2ccccc2)sc2c1CCC2

Physicochemical Properties	
Formula	C20H22N4OS
Molecular weight	366.48 g/mol
Num. heavy atoms	26
Num. arom. heavy atoms	11
Fraction Csp3	0.40
Num. rotatable bonds	5
Num. H-bond acceptors	3
Num. H-bond donors	1
Molar Refractivity	111.75
TPSA	87.61 Å²

Lipophilicity	
Log $P_{o/w}$ (iLOGP)	3.03
Log $P_{o/w}$ (XLOGP3)	3.73
Log $P_{o/w}$ (WLOGP)	1.92
Log $P_{o/w}$ (MLOGP)	1.73
Log $P_{o/w}$ (SILICOS-IT)	3.81
Consensus Log $P_{o/w}$	2.84

Water Solubility	
Log *S* (ESOL)	-4.45
Solubility	1.31e-02 mg/ml ; 3.59e-05 mol/l
Class	Moderately soluble
Log *S* (Ali)	-5.26
Solubility	2.01e-03 mg/ml ; 5.47e-06 mol/l
Class	Moderately soluble
Log *S* (SILICOS-IT)	-5.24
Solubility	2.13e-03 mg/ml ; 5.81e-06 mol/l
Class	Moderately soluble

Pharmacokinetics	
GI absorption	High
BBB permeant	No
P-gp substrate	No
CYP1A2 inhibitor	Yes
CYP2C19 inhibitor	Yes
CYP2C9 inhibitor	Yes
CYP2D6 inhibitor	Yes
CYP3A4 inhibitor	Yes
Log K_p (skin permeation)	-5.89 cm/s

Druglikeness	
Lipinski	Yes; 0 violation
Ghose	Yes
Veber	Yes
Egan	Yes
Muegge	Yes
Bioavailability Score	0.55

Medicinal Chemistry	
PAINS	0 alert
Brenk	0 alert
Leadlikeness	No; 2 violations: MW>350, XLOGP3>3.5
Synthetic accessibility	3.48

Tiamulin

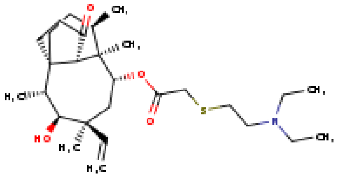

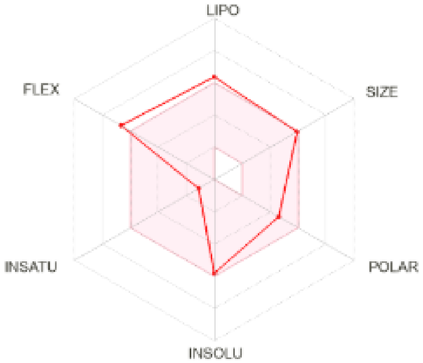

SMILES	CCN(CCSCC(=O)O[C@@H]1C[C@@](C)(C=C)[C@@H](O)[C@@H]([C@]23[C@H]([C@@]1(C)[C@H](C)CC2)C(=O)CC3)C)CC
**Physicochemical Properties**	
Formula	C28H47NO4S
Molecular weight	493.74 g/mol
Num. heavy atoms	34
Num. arom. heavy atoms	0
Fraction Csp3	0.86
Num. rotatable bonds	10
Num. H-bond acceptors	5
Num. H-bond donors	1
Molar Refractivity	142.25
TPSA	92.14 Å²
**Lipophilicity**	
Log $P_{o/w}$ (iLOGP)	4.05
Log $P_{o/w}$ (XLOGP3)	5.65
Log $P_{o/w}$ (WLOGP)	4.97
Log $P_{o/w}$ (MLOGP)	3.66
Log $P_{o/w}$ (SILICOS-IT)	5.28
Consensus Log $P_{o/w}$	4.72

**Water Solubility**	
Log *S* (ESOL)	-5.80
Solubility	7.81e-04 mg/ml ; 1.58e-06 mol/l
Class	Moderately soluble
Log *S* (Ali)	-7.35
Solubility	2.21e-05 mg/ml ; 4.48e-08 mol/l
Class	Poorly soluble
Log *S* (SILICOS-IT)	-5.65
Solubility	1.10e-03 mg/ml ; 2.23e-06 mol/l
Class	Moderately soluble
**Pharmacokinetics**	
GI absorption	High
BBB permeant	No
P-gp substrate	No
CYP1A2 inhibitor	No
CYP2C19 inhibitor	No
CYP2C9 inhibitor	No
CYP2D6 inhibitor	No
CYP3A4 inhibitor	Yes
Log K_p (skin permeation)	-5.30 cm/s
**Druglikeness**	
Lipinski	Yes; 0 violation
Ghose	No; 3 violations: MW>480, MR>130, #atoms>70
Veber	Yes
Egan	Yes
Muegge	No; 1 violation: XLOGP3>5
Bioavailability Score	0.55
**Medicinal Chemistry**	
PAINS	0 alert
Brenk	1 alert: isolated_alkene
Leadlikeness	No; 3 violations: MW>350, Rotors>7, XLOGP3>3.5
Synthetic accessibility	6.58

Figure S4: Clustal alignment of glutamate racemase (MurI) amino acid sequences from isolates of *B. pilosicoli* in Genbank

```
MEI0580414.1        MKINSMSIAVFDSGFGGISVLKKLLDILPNENYIYLGDNCNIPYGDKSKEEITQLSIKIL       60
WP_335762868.1      MKINSMSIAVFDSGFGGISVLKKLLDILPNENYIYLGDNCNIPYGDKSKEEITQLSIKIL       60
TXJ46490.1          MNTSSKHIAVFDSGFGGISVLKKLLDMLPNENYIYLGDNHNIPYGDKSKEEITQLSIKIL       60
WP_147730525.1      MNTSSKHIAVFDSGFGGISVLKKLLDMLPNENYIYLGDNHNIPYGDKSKEEITQLSIKIL       60
MEI0531133.1        MSVLSKPIAVFDSGFGGVSVLKKLLNMLPNENYIYLGDNSNIPYGDKSKDEINKLSIKIL       60
WP_335784214.1      MSVLSKPIAVFDSGFGGVSVLKKLLNMLPNENYIYLGDNSNIPYGDKSKDEINKLSIKIL       60
MEI0611424.1        MSISSKPIAVFDSGFGGISVLKKLLDILPNENYIYLGDNHNIPYGDKSKEEITQLSIKIL       60
WP_335764741.1      MSISSKPIAVFDSGFGGISVLKKLLDILPNENYIYLGDNHNIPYGDKSKEEITQLSIKIL       60
MBW5397281.1        MSISSKPIAVFDSGFGGISVLKKLLNILPNENYIYLGDNHNIPYGDKSKEEITQLSIKIL       60
WP_219709157.1      MSISSKPIAVFDSGFGGISVLKKLLNILPNENYIYLGDNHNIPYGDKSKEEITQLSIKIL       60
AFR70668.1          MSISSKPIAVFDSGFGGISVLKKLLNILPNENYIYLGDNHNIPYGDKSKEEITQLSIKIL       60
WP_014935973.1      MSISSKPIAVFDSGFGGISVLKKLLNILPNENYIYLGDNHNIPYGDKSKEEITQLSIKIL       60
WP_286032723.1      MSISSKPIAVFDSGFGGISVLKKLLNILPNENYIYIGDNHNIPYGDKSKEEITQLSIKIL       60
WP_281818569.1      MSISSKPIAVFDSGFGGISVLKKLLNILPNENYIYLGDNHNIPYGDKSKEEITQLSIKIL       60
WP_157149352.1      MSISSKPIAVFDSGFGGISVLKKLLNILPNENYIYLGDNHNIPYGDKSKEEITQLSIKIL       60
SUW07777.1          MSISSKPIAVFDSGFGGISVLKKLLNILPNENYIYLGDNHNIPYGDKSKEEITQLSIKIL       60
WP_115589036.1      MSISSKPIAVFDSGFGGISVLKKLLNILPNENYIYLGDNHNIPYGDKSKEEITQLSIKIL       60
MEI0794885.1        MSISSKPIAVFDSGFGGISVLKKLLNILPNENYIYLGDNHNIPYGDKSKEEITQLSIKIL       60
WP_335770947.1      MSISSKPIAVFDSGFGGISVLKKLLNILPNENYIYLGDNHNIPYGDKSKEEITQLSIKIL       60
MEI0561566.1        MSISSKPIAVFDSGFGGISVLKKLLNILPNENYIYLGDNHNIPYGDKSKEEITQLSIKIL       60
MEI0628508.1        MSISSKPIAVFDSGFGGISVLKKLLNILPNENYIYLGDNHNIPYGDKSKEEITQLSIKIL       60
WP_157145903.1      MSISSKPIAVFDSGFGGISVLKKLLNILPNENYIYLGDNHNIPYGDKSKEEITQLSIKIL       60
WIH88613.1          MSISSKPIAVFDSGFGGISVLKKLLNILPNENYIYLGDNHNIPYGDKSKEEITQLSIKIL       60
WP_284635581.1      MSISSKPIAVFDSGFGGISVLKKLLNILPNENYIYLGDNHNIPYGDKSKEEITQLSIKIL       60
MBW5382395.1        MSISSKPIAVFDSGFGGISVLKKLLNILPNENYIYLGDNHNIPYGDKSKEEITQLSIKIL       60
WP_219699457.1      MSISSKPIAVFDSGFGGISVLKKLLNILPNENYIYLGDNHNIPYGDKSKEEITQLSIKIL       60
MBW5398612.1        MSISSKPIAVFDSGFGGISVLKKLLNILPNENYIYLGDNHNIPYGDKSKEEITQLSIKIL       60
WIH81843.1          MSISSKPIAVFDSGFGGISVLKKLLNILPNENYIYLGDNHNIPYGDKSKEEITQLSIKIL       60
WIH86281.1          MSISSKPIAVFDSGFGGISVLKKLLNILPNENYIYLGDNHNIPYGDKSKEEITQLSIKIL       60
WIH90881.1          MSISSKPIAVFDSGFGGISVLKKLLNILPNENYIYLGDNHNIPYGDKSKEEITQLSIKIL       60
WIH93172.1          MSISSKPIAVFDSGFGGISVLKKLLNILPNENYIYLGDNHNIPYGDKSKEEITQLSIKIL       60
```

```
WIH95462.1          MSISSKPIAVFDSGFGGISVLKKLLNILPNENYIYLGDNHNIPYGDKSKEEITQLSIKIL       60
WP_157143181.1      MSISSKPIAVFDSGFGGISVLKKLLNILPNENYIYLGDNHNIPYGDKSKEEITQLSIKIL       60
BpP43/6/78          MSISSKPIAVFDSGFGGISVLKKLLNILPNENYIYLGDNHNIPYGDKSKEEITQLSIKIL       60
WIH84105.1          MSISSKPIAVFDSGFGGISVLKKLLNILPNENYIYLGDNHNIPYGDKSKEEITQLSIKIL       60
WP_015274086.1      MSISSKPIAVFDSGFGGISVLKKLLNILPNENYIYLGDNHNIPYGDKSKEEITQLSIKIL       60
ADK30055.1          MSISSKPIAVFDSGFGGISVLKKLLNILPNENYIYLGDNHNIPYGDKSKEEITQLSIKIL       60
CCG56942.1          MSISSKPIAVFDSGFGGISVLKKLLNILPNENYIYLGDNHNIPYGDKSKEEITQLSIKIL       60
PLV60746.1          MSISSKPIAVFDSGFGGISVLKKLLNILPNENYIYLGDNHNIPYGDKSKEEITQLSIKIL       60
MEI0609641.1        MSISSKPIAVFDSGFGGISVLKKLLNILPNENYIYLGDNHNIPYGDKSKEEITQLSIKIL       60
MEI0541833.1        MSISSKPIAVFDSGFGGISVLKKLLNILPNENYIYLGDNHNIPYGDKSKEEITQLSIKIL       60
MEI0822658.1        MSISSKPIAVFDSGFGGISVLKKLLNILPNENYIYLGDNHNIPYGDKSKEEITQLSIKIL       60
MEI0619229.1        MSISSKPIAVFDSGFGGISVLKKLLNILPNENYIYLGDNHNIPYGDKSKEEITQLSIKIL       60
MEI0844465.1        MSISSKPIAVFDSGFGGISVLKKLLNILPNENYIYLGDNHNIPYGDKSKEEITQLSIKIL       60
MEI0684780.1        MSISSKPIAVFDSGFGGISVLKKLLNILPNENYIYLGDNHNIPYGDKSKEEITQLSIKIL       60
MEI0820803.1        MSISSKPIAVFDSGFGGISVLKKLLNILPNENYIYLGDNHNIPYGDKSKEEITQLSIKIL       60
SUW05994.1          MSISSKPIAVFDSGFGGISVLKKLLNILPNENYIYLGDNHNIPYGDKSKEEITQLSIKIL       60
WP_013243010.1      MSISSKPIAVFDSGFGGISVLKKLLNILPNENYIYLGDNHNIPYGDKSKEEITQLSIKIL       60
                    *.  *  **********:*******::********:*** *********:**.:******

MEI0580414.1        DFLIKQNCKMAVIACNTITASSYDILKERYN-IPIIEVISNGVEDIIDNTKNNNVSIMAT       119
WP_335762868.1      DFLIKQNCKMAVIACNTITASSYDILKERYN-IPIIEVISNGVEDIIDNTKNNNVSIMAT       119
TXJ46490.1          DFLIKQDCKMAVIACNTITASSYDILKEKYN-IPIIETISHGVEDIIDNTKNNNISIMAT       119
WP_147730525.1      DFLIKQDCKMAVIACNTITASSYDILKEKYN-IPIIETISHGVEDIIDNTKNNNISIMAT       119
MEI0531133.1        DFLVKQNCKMAVIACNTITASSYDILKEKYNNIPIIEIISNGVEDIIDNTKNNNISIMAT       120
WP_335784214.1      DFLVKQNCKMAVIACNTITASSYDILKEKYNNIPIIEIISNGVEDIIDNTKNNNISIMAT       120
MEI0611424.1        DFLIKQNCKMAVIACNTITASSYDILKEKYN-IPIIEIISNGVEDIIDNTKNNNISIMAT       119
WP_335764741.1      DFLIKQNCKMAVIACNTITASSYDILKEKYN-IPIIEIISNGVEDIIDNTKNNNISIMAT       119
MBW5397281.1        DFLIKQDCKMAVIACNTITASSYDFLKEKYD-IPIIEIISNGVEDIIDNTKNNNISIMAT       119
WP_219709157.1      DFLIKQDCKMAVIACNTITASSYDFLKEKYD-IPIIEIISNGVEDIIDNTKNNNISIMAT       119
AFR70668.1          DFLIKQNCKMAVIACNTITASSYDVLKEKYN-IPIIEIISNGVEDIIDNTKNNNISIMAT       119
WP_014935973.1      DFLIKQNCKMAVIACNTITASSYDVLKEKYN-IPIIEIISNGVEDIIDNTKNNNISIMAT       119
WP_286032723.1      DFLIKQNCKMAVIACNTITASSYDVLKEKYN-IPIIEIISNGVEDIIDNTKNNNISIMAT       119
WP_281818569.1      DFLIKQNCKMAVIACNTITASSYDVLKEKYN-IPIIEIISNGVEDIIDNTKNNNISIMAT       119
```

WP_157149352.1	DFLIKQNCKMAVIACNTITASSYDILKEKYN-IPIIEIISNGVEDIIDNTKNNNISIMAT	119
SUW07777.1	DFLIKQNCKMAVIACNTITASSYDVLKEKYN-IPIIEIISNGVEDIIDNTKNNNISIMAT	119
WP_115589036.1	DFLIKQNCKMAVIACNTITASSYDVLKEKYN-IPIIEIISNGVEDIIDNTKNNNISIMAT	119
MEI0794885.1	DFLIKQNCKMAVIACNTITASSYDVLKEKYN-IPIIEIISNGVEDIIDNTKNNNISIMAT	119
WP_335770947.1	DFLIKQNCKMAVIACNTITASSYDVLKEKYN-IPIIEIISNGVEDIIDNTKNNNISIMAT	119
MEI0561566.1	DFLIKQNCKMAVIACNTITASSYDVLKEKYN-IPIIEIISNGVEDIIDNTKNNNISIMAT	119
MEI0628508.1	DFLIKQNCKMAVIACNTITASSYDVLKEKYN-IPIIEIISNGVEDIIDNTKNNNISIMAT	119
WP_157145903.1	DFLIKQNCKMAVIACNTITASSYDVLKEKYN-IPIIEIISNGVEDIIDNTKNNNISIMAT	119
WIH88613.1	DFLIKQNCKMAVIACNTITASSYDVLKEKYN-IPIIEIISNGVEDIIDNTKNNNISIMAT	119
WP_284635581.1	DFLIKQNCKMAVIACNTITASSYDVLKEKYN-IPIIEIISNGVEDIIDNTKNNNISIMAT	119
MBW5382395.1	DFLIKQNCKMAVIACNTITASSYDVLKEKYN-IPIIEIISNGVEDIIDNTKNNNISIMAT	119
WP_219699457.1	DFLIKQNCKMAVIACNTITASSYDVLKEKYN-IPIIEIISNGVEDIIDNTKNNNISIMAT	119
MBW5398612.1	DFLIKQNCKMAVIACNTITASSYDVLKEKYN-IPIIEIISNGVEDIIDNTKNNNISIMAT	119
WIH81843.1	DFLIKQNCKMAVIACNTITASSYDVLKEKYN-IPIIEIISNGVEDIIDNTKNNNISIMAT	119
WIH86281.1	DFLIKQNCKMAVIACNTITASSYDVLKEKYN-IPIIEIISNGVEDIIDNTKNNNISIMAT	119
WIH90881.1	DFLIKQNCKMAVIACNTITASSYDVLKEKYN-IPIIEIISNGVEDIIDNTKNNNISIMAT	119
WIH93172.1	DFLIKQNCKMAVIACNTITASSYDVLKEKYN-IPIIEIISNGVEDIIDNTKNNNISIMAT	119
WIH95462.1	DFLIKQNCKMAVIACNTITASSYDVLKEKYN-IPIIEIISNGVEDIIDNTKNNNISIMAT	119
WP_157143181.1	DFLIKQNCKMAVIACNTITASSYDVLKEKYN-IPIIEIISNGVEDIIDNTKNNNISIMAT	119
BpP43/6/78	DFLIKQNCKMAVIACNTITASSYDVLKDKYN-IPIIEIISNGVEDIIDNTKNNNISIMAT	119
WIH84105.1	DFLIKQNCKMAVIACNTITASSYDVLKDKYN-IPIIEIISNGVEDIIDNTKNNNISIMAT	119
WP_015274086.1	DFLIKQNCKMAVIACNTITASSYDVLKDKYN-IPIIEIISNGVEDIIDNTKNNNISIMAT	119
ADK30055.1	DFLIKQNCKMAVIACNTITASSYDVLKEKYN-IPIIEIISNGVEDIIDNTKNNNISIMAT	119
CCG56942.1	DFLIKQNCKMAVIACNTITASSYDVLKEKYN-IPIIEIISNGVEDIIDNTKNNNISIMAT	119
PLV60746.1	DFLIKQNCKMAVIACNTITASSYDVLKEKYN-IPIIEIISNGVEDIIDNTKNNNISIMAT	119
MEI0609641.1	DFLIKQNCKMAVIACNTITASSYDVLKEKYN-IPIIEIISNGVEDIIDNTKNNNISIMAT	119
MEI0541833.1	DFLIKQNCKMAVIACNTITASSYDVLKEKYN-IPIIEIISNGVEDIIDNTKNNNISIMAT	119
MEI0822658.1	DFLIKQNCKMAVIACNTITASSYDVLKEKYN-IPIIEIISNGVEDIIDNTKNNNISIMAT	119
MEI0619229.1	DFLIKQNCKMAVIACNTITASSYDVLKEKYN-IPIIEIISNGVEDIIDNTKNNNISIMAT	119
MEI0844465.1	DFLIKQNCKMAVIACNTITASSYDVLKEKYN-IPIIEIISNGVEDIIDNTKNNNISIMAT	119
MEI0684780.1	DFLIKQNCKMAVIACNTITASSYDVLKEKYN-IPIIEIISNGVEDIIDNTKNNNISIMAT	119
MEI0820803.1	DFLIKQNCKMAVIACNTITASSYDVLKEKYN-IPIIEIISNGVEDIIDNTKNNNISIMAT	119
SUW05994.1	DFLIKQNCKMAVIACNTITASSYDVLKEKYN-IPIIEIISNGVEDIIDNTKNNNISIMAT	119

```
WP_013243010.1      DFLIKQNCKMAVIACNTITASSYDVLKEKYN-IPIIEIISNGVEDIIDNTKNNNISIMAT      119
                    ***:**:*****************.**::*: ***** **:*************:*****

MEI0580414.1        EFTVHSNVYYDKIFNYNKKIKVTQVACQKLCPMIENNWCSYDDRLNVLEEYVKRIDDDSD      179
WP_335762868.1      EFTVHSNVYYDKIFNYNKKIKVTQVACQKLCPMIENNWCSYDDRLNVLEEYVKRIDDDSD      179
TXJ46490.1          EFTVHSNMYHDKILDYNKKMKVTQVACQKLCPMIENNWYSYDDRFKVLEEYIKKIDDNSD      179
WP_147730525.1      EFTVHSNMYHDKILDYNKKMKVTQVACQKLCPMIENNWYSYDDRFKVLEEYIKKIDDNSD      179
MEI0531133.1        EFTVHSNIYHDKILHYNDKIKVTQVACQKLCPMIENNWYSYDDRLKVLEEYVKKIDDNSD      180
WP_335784214.1      EFTVHSNIYHDKILHYNDKIKVTQVACQKLCPMIENNWYSYDDRLKVLEEYVKKIDDNSD      180
MEI0611424.1        EFTVHSNMYNDKILNYNDKIKVTQVACQKLCPMIENNWYSYDNRLKVLEEYVKKIDDNSD      179
WP_335764741.1      EFTVHSNMYNDKILNYNDKIKVTQVACQKLCPMIENNWYSYDNRLKVLEEYVKKIDDNSD      179
MBW5397281.1        EFTVHSNMYHDKILNYNDKIKVTQVACQKLCPMIENNWYSYDDRLKVLEEYVKKIDDNSD      179
WP_219709157.1      EFTVHSNMYHDKILNYNDKIKVTQVACQKLCPMIENNWYSYDDRLKVLEEYVKKIDDNSD      179
AFR70668.1          EFTVHSNMYHDKILDYNDKIKVTQVACQKLCPMIENNWYSYDDRILVLEEYVKKIDDNSD      179
WP_014935973.1      EFTVHSNMYHDKILDYNDKIKVTQVACQKLCPMIENNWYSYDDRILVLEEYVKKIDDNSD      179
WP_286032723.1      EFTVHSNMYHDKILDYNDKIKVTQVACQKLCPMIENNWYSYDDRILVLEEYVKKIDDNSD      179
WP_281818569.1      EFTVHSNMYHDKILDYNDKIKVTQVACQKLCPMIENNWYSYDDRILVLEEYVKKIDDNSD      179
WP_157149352.1      EFTVHSNMYHDKILDYNDKIKVTQVACQKLCPMIENNWYSYDDRILVLEEYVKKIDDNSD      179
SUW07777.1          EFTVHSNMYHDKIFDYNDKIKVTQVACQKLCPMIENNWYSYDDRILVLEEYVKKIDDNSD      179
WP_115589036.1      EFTVHSNMYHDKIFDYNDKIKVTQVACQKLCPMIENNWYSYDDRILVLEEYVKKIDDNSD      179
MEI0794885.1        EFTVHSNMYHDKILDYNDKIKVTQVACQKLCPMIENNWYSYDDRILVLEEYVKKIDDNSD      179
WP_335770947.1      EFTVHSNMYHDKILDYNDKIKVTQVACQKLCPMIENNWYSYDDRILVLEEYVKKIDDNSD      179
MEI0561566.1        EFTVHSNMYHDKILDYNDKIKVTQVACQKLCPMIENNWYSYDDRILVLEEYVKKIDDNSD      179
MEI0628508.1        EFTVHSNMYHDKILDYNDKIKVTQVACQKLCPMIENNWYSYDDRILVLEEYVKKIDDNSD      179
WP_157145903.1      EFTVHSNMYHDKILDYNDKIKVTQVACQKLCPMIENNWYSYDDRILVLEEYVKKIDDNSD      179
WIH88613.1          EFTVHSNMYHDKILDYNDKIKVTQVACQKLCPMIENNWYSYDDRILVLEEYIKKIDDNSD      179
WP_284635581.1      EFTVHSNMYHDKILDYNDKIKVTQVACQKLCPMIENNWYSYDDRILVLEEYIKKIDDNSD      179
MBW5382395.1        EFTVHSNMYHDKILDYNDKIKVTQVACQKLCPMIENNWYNYDDRILVLEEYVKKIDDNSD      179
WP_219699457.1      EFTVHSNMYHDKILDYNDKIKVTQVACQKLCPMIENNWYNYDDRILVLEEYVKKIDDNSD      179
MBW5398612.1        EFTVHSNMYHDKILDYNDKIKVTQVACQKLCPMIENNWYSYDDRILVLEEYVKKIDDNSD      179
WIH81843.1          EFTVHSNMYHDKILDYNDKIKVTQVACQKLCPMIENNWYSYDDRILVLEEYVKKIDDNSD      179
WIH86281.1          EFTVHSNMYHDKILDYNDKIKVTQVACQKLCPMIENNWYSYDDRILVLEEYVKKIDDNSD      179
WIH90881.1          EFTVHSNMYHDKILDYNDKIKVTQVACQKLCPMIENNWYSYDDRILVLEEYVKKIDDNSD      179
```

```
WIH93172.1          EFTVHSNMYHDKILDYNDKIKVTQVACQKLCPMIENNWYSYDDRILVLEEYVKKIDDNSD       179
WIH95462.1          EFTVHSNMYHDKILDYNDKIKVTQVACQKLCPMIENNWYSYDDRILVLEEYVKKIDDNSD       179
WP_157143181.1      EFTVHSNMYHDKILDYNDKIKVTQVACQKLCPMIENNWYSYDDRILVLEEYVKKIDDNSD       179
BpP43/6/78          EFTVHSNMYHDKILDYNDKIKVTQVACQKLCPMIENNWYSYDDRILVLEEYVKKIDDNSD       179
WIH84105.1          EFTVHSNMYHDKILDYNDKIKVTQVACQKLCPMIENNWYSYDDRILVLEEYVKKIDDNSD       179
WP_015274086.1      EFTVHSNMYHDKILDYNDKIKVTQVACQKLCPMIENNWYSYDDRILVLEEYVKKIDDNSD       179
ADK30055.1          EFTVHSNMYHDKILDYNDKIKVTQVACQKLCPMIENNWYSYDDRILVLEEYVKKIDDNSD       179
CCG56942.1          EFTVHSNMYHDKILDYNDKIKVTQVACQKLCPMIENNWYSYDDRILVLEEYVKKIDDNSD       179
PLV60746.1          EFTVHSNMYHDKILDYNDKIKVTQVACQKLCPMIENNWYSYDDRILVLEEYVKKIDDNSD       179
MEI0609641.1        EFTVHSNMYHDKILDYNDKIKVTQVACQKLCPMIENNWYSYDDRILVLEEYVKKIDDNSD       179
MEI0541833.1        EFTVHSNMYHDKILDYNDKIKVTQVACQKLCPMIENNWYSYDDRILVLEEYVKKIDDNSD       179
MEI0822658.1        EFTVHSNMYHDKILDYNDKIKVTQVACQKLCPMIENNWYSYDDRILVLEEYVKKIDDNSD       179
MEI0619229.1        EFTVHSNMYHDKILDYNDKIKVTQVACQKLCPMIENNWYSYDDRILVLEEYVKKIDDNSD       179
MEI0844465.1        EFTVHSNMYHDKILDYNDKIKVTQVACQKLCPMIENNWYSYDDRILVLEEYVKKIDDNSD       179
MEI0684780.1        EFTVHSNMYHDKILDYNDKIKVTQVACQKLCPMIENNWYSYDDRILVLEEYVKKIDDNSD       179
MEI0820803.1        EFTVHSNMYHDKILDYNDKIKVTQVACQKLCPMIENNWYSYDDRILVLEEYVKKIDDNSD       179
SUW05994.1          EFTVHSNMYHDKILDYNDKIKVTQVACQKLCPMIENNWYSYDDRILVLEEYVKKIDDNSD       179
WP_013243010.1      EFTVHSNMYHDKILDYNDKIKVTQVACQKLCPMIENNWYSYDDRILVLEEYVKKIDDNSD       179
                    *******:* ***:.**.*:****************** .**:*: *****:*:***:**

MEI0580414.1        TLLLACTHYPFIMDDIKDVIDRKKTNIKNIIDPSQKIALSIKKYLIDNNLVNTSGGNLKF       239
WP_335762868.1      TLLLACTHYPFIMDDIKDVIDRKKTNIKNIIDPSQKIALSIKKYLIDNNLVNTSGGNLKF       239
TXJ46490.1          TLLLACTHYPLIMDDIKAVVNIKKTNIKNIIDPSTKIALSIKKYLLENNLINNSGGSLKF       239
WP_147730525.1      TLLLACTHYPLIMDDIKAVVNIKKTNIKNIIDPSTKIALSIKKYLLENNLINNSGGSLKF       239
MEI0531133.1        TLLLACTHYPFIIDDIKSVVNNKKTNIKNIIDPSTKIALSIKKYIIDNNLANTSGGHLKF       240
WP_335784214.1      TLLLACTHYPFIIDDIKSVVNNKKTNIKNIIDPSTKIALSIKKYIIDNNLANTSGGHLKF       240
MEI0611424.1        TLLLACTHYPFIIDDIKSVVDRKKTNIKNIIDPSNKMSLSIKKYLIDNNLVNNSGGKLKF       239
WP_335764741.1      TLLLACTHYPFIIDDIKSVVDRKKTNIKNIIDPSNKMSLSIKKYLIDNNLVNNSGGKLKF       239
MBW5397281.1        TLLLACTHYPFIIDDIKYVVNNKKTNIKNIIDPSTKIALSIKKYLIDNNLVNNSGGRLKF       239
WP_219709157.1      TLLLACTHYPFIIDDIKYVVNNKKTNIKNIIDPSTKIALSIKKYLIDNNLVNNSGGRLKF       239
AFR70668.1          TLLLACTHYPFIIDDIKSVVNRKKTNIKNIIDPSTKIALSIKKYLIDNNLLNTSGGKLKF       239
WP_014935973.1      TLLLACTHYPFIIDDIKSVVNRKKTNIKNIIDPSTKIALSIKKYLIDNNLLNTSGGKLKF       239
WP_286032723.1      TLLLACTHYPFIIDDIKSVVNRKKTNIKNIIDPSTKIALSIKKYLIDNNLLNTSGGKLKF       239
```

WP_281818569.1	TLLLACTHYPFIIDDIKSVVNRKKANIKNIIDPSTKIALSIKKYLIDNNLLNTSGGKLKF	239
WP_157149352.1	TLLLACTHYPFIIDDIKSVVNRKKTNIKNIIDPSTKIALSIKKYLIDNNLLNTSGGKLKF	239
SUW07777.1	TLLLACTHYPFIIDDIKSVVNRKKTNIKNIIDPSTKIALSIKKYLIDNNLLNTSGGKLKF	239
WP_115589036.1	TLLLACTHYPFIIDDIKSVVNRKKTNIKNIIDPSTKIALSIKKYLIDNNLLNTSGGKLKF	239
MEI0794885.1	TLLLACTHYPFIIDDIKSVVNRKKANIKNIIDPSTKIALSIKKYLIDNILLNTSGGKLKF	239
WP_335770947.1	TLLLACTHYPFIIDDIKSVVNRKKANIKNIIDPSTKIALSIKKYLIDNILLNTSGGKLKF	239
MEI0561566.1	TLLLACTHYPFIIDDIKSVVNRKKTNIKNIIEPSTKIALSIKKYLIDNNLLNTSGGKLKF	239
MEI0628508.1	TLLLACTHYPFIIDDIKSVVNRKKTNIKNIIEPSTKIALSIKKYLIDNNLLNTSGGKLKF	239
WP_157145903.1	TLLLACTHYPFIIDDIKSVVNRKKTNIKNIIEPSTKIALSIKKYLIDNNLLNTSGGKLKF	239
WIH88613.1	TLLLACTHYPFIIDDIKSVVNRKKANIKNIIDPSTKIALSIKKYLIDNNLLNTSGGKLKF	239
WP_284635581.1	TLLLACTHYPFIIDDIKSVVNRKKANIKNIIDPSTKIALSIKKYLIDNNLLNTSGGKLKF	239
MBW5382395.1	TLLLACTHYPFIIDDIKSVVNRKKTNIKNIIDPSTKIALSIKKYLIDNNLLNTSGGKLKF	239
WP_219699457.1	TLLLACTHYPFIIDDIKSVVNRKKTNIKNIIDPSTKIALSIKKYLIDNNLLNTSGGKLKF	239
MBW5398612.1	TLLLACTHYPFIIDDIKSVVNRKKANIKNIIDPSTKIALSIKKYLIDNNLLNTSGGKLKF	239
WIH81843.1	TLLLACTHYPFIIDDIKSVVNRKKANIKNIIDPSTKIALSIKKYLIDNNLLNTSGGKLKF	239
WIH86281.1	TLLLACTHYPFIIDDIKSVVNRKKANIKNIIDPSTKIALSIKKYLIDNNLLNTSGGKLKF	239
WIH90881.1	TLLLACTHYPFIIDDIKSVVNRKKANIKNIIDPSTKIALSIKKYLIDNNLLNTSGGKLKF	239
WIH93172.1	TLLLACTHYPFIIDDIKSVVNRKKANIKNIIDPSTKIALSIKKYLIDNNLLNTSGGKLKF	239
WIH95462.1	TLLLACTHYPFIIDDIKSVVNRKKANIKNIIDPSTKIALSIKKYLIDNNLLNTSGGKLKF	239
WP_157143181.1	TLLLACTHYPFIIDDIKSVVNRKKANIKNIIDPSTKIALSIKKYLIDNNLLNTSGGKLKF	239
BpP43/6/78	TLLLACTHYPFIIDDIKSVVNRKKTNIKNIIDPSTKIALSIKKYLIDNNLLNTSGGKLKF	239
WIH84105.1	TLLLACTHYPFIIDDIKSVVNRKKTNIKNIIDPSTKIALSIKKYLIDNNLLNTSGGKLKF	239
WP_015274086.1	TLLLACTHYPFIIDDIKSVVNRKKTNIKNIIDPSTKIALSIKKYLIDNNLLNTSGGKLKF	239
ADK30055.1	TLLLACTHYPFIIDDIKSVVNRKKTNIKNIIDPSTKIALSIKKYLIDNNLLNTSGGKLKF	239
CCG56942.1	TLLLACTHYPFIIDDIKSVVNRKKTNIKNIIDPSTKIALSIKKYLIDNNLLNTSGGKLKF	239
PLV60746.1	TLLLACTHYPFIIDDIKSVVNRKKTNIKNIIDPSTKIALSIKKYLIDNNLLNTSGGKLKF	239
MEI0609641.1	TLLLACTHYPFIIDDIKSVVNRKKTNIKNIIDPSTKIALSIKKYLIDNNLLNTSGGKLKF	239
MEI0541833.1	TLLLACTHYPFIIDDIKSVVNRKKTNIKNIIDPSTKIALSIKKYLIDNNLLNTSGGKLKF	239
MEI0822658.1	TLLLACTHYPFIIDDIKSVVNRKKTNIKNIIDPSTKIALSIKKYLIDNNLLNTSGGKLKF	239
MEI0619229.1	TLLLACTHYPFIIDDIKSVVNRKKTNIKNIIDPSTKIALSIKKYLIDNNLLNTSGGKLKF	239
MEI0844465.1	TLLLACTHYPFIIDDIKSVVNRKKTNIKNIIDPSTKIALSIKKYLIDNNLLNTSGGKLKF	239
MEI0684780.1	TLLLACTHYPFIIDDIKSVVNRKKTNIKNIIDPSTKIALSIKKYLIDNNLLNTSGGKLKF	239
MEI0820803.1	TLLLACTHYPFIIDDIKSVVNRKKTNIKNIIDPSTKIALSIKKYLIDNNLLNTSGGKLKF	239

```
SUW05994.1          TLLLACTHYPFIIDDIKSVVNRKKTNIKNIIDPSTKIALSIKKYLIDNNLLNTSGGKLKF       239
WP_013243010.1      TLLLACTHYPFIIDDIKSVVNRKKTNIKNIIDPSTKIALSIKKYLIDNNLLNTSGGKLKF       239
                    **********:*:**** *:: **:******:**:*::******:::* * *.*** ***

MEI0580414.1        FTTGNKKDFNDFVSKYIKIDYELERIVL   267
WP_335762868.1      FTTGNKKDFNDFVSKYIKIDYELERIVL   267
TXJ46490.1          FTTGSKKDFNDFVSIYIKNNYELERVVL   267
WP_147730525.1      FTTGSKKDFNDFVSIYIKNNYELERVVL   267
MEI0531133.1        FTTGNKKDFNDFISRYIKINYELERIVL   268
WP_335784214.1      FTTGNKKDFNDFISRYIKINYELERIVL   268
MEI0611424.1        FTTGDKKDFNDFVSRYIKINYELERIVL   267
WP_335764741.1      FTTGDKKDFNDFVSRYIKINYELERIVL   267
MBW5397281.1        FTTGDKKDFNDFVSRYVKINYELERIVL   267
WP_219709157.1      FTTGDKKDFNDFVSRYVKINYELERIVL   267
AFR70668.1          FTTGDKKILMILY---LDI---------   255
WP_014935973.1      FTTGDKKILMILY---LDI---------   255
WP_286032723.1      FTTGDKKDFNDFVSRYIKINYELERIVL   267
WP_281818569.1      FTTGNKKDFNDFVSRYIKINYELERIVL   267
WP_157149352.1      FTTGDKKDFNDFVSRYIKINYELERIVL   267
SUW07777.1          FTTGDKKDFNDFVSRYIKINYELERIVL   267
WP_115589036.1      FTTGDKKDFNDFVSRYIKINYELERIVL   267
MEI0794885.1        FTTGDKKDFNDFVSRYIKINYELERIVL   267
WP_335770947.1      FTTGDKKDFNDFVSRYIKINYELERIVL   267
MEI0561566.1        FTTGDKKDFNDFVSRYIKINYELERIVL   267
MEI0628508.1        FTTGDKKDFNDFVSRYIKINYELERIVL   267
WP_157145903.1      FTTGDKKDFNDFVSRYIKINYELERIVL   267
WIH88613.1          FTTGDKKDFNDFVSRYIKINYELERIVL   267
WP_284635581.1      FTTGDKKDFNDFVSRYIKINYELERIVL   267
MBW5382395.1        FTTGDKKDFNDFVSRYIKINYELERIVL   267
WP_219699457.1      FTTGDKKDFNDFVSRYIKINYELERIVL   267
MBW5398612.1        FTTGDKKDFNDFVSRYIKINYELERIVL   267
WIH81843.1          FTTGDKKDFNDFVSRYIKINYELERIVL   267
WIH86281.1          FTTGDKKDFNDFVSRYIKINYELERIVL   267
```

```
WIH90881.1          FTTGDKKDFNDFVSRYIKINYELERIVL   267
WIH93172.1          FTTGDKKDFNDFVSRYIKINYELERIVL   267
WIH95462.1          FTTGDKKDFNDFVSRYIKINYELERIVL   267
WP_157143181.1      FTTGDKKDFNDFVSRYIKINYELERIVL   267
BpP43/6/78          FTTGDKKDFNDFVSRYIKINYELERIVL   267
WIH84105.1          FTTGDKKDFNDFVSRYIKINYELERIVL   267
WP_015274086.1      FTTGDKKDFNDFVSRYIKINYELERIVL   267
ADK30055.1          FTTGDKKDFNDFVSRYIKINYELERIVL   267
CCG56942.1          FTTGDKKDFNDFVSRYIKINYELERIVL   267
PLV60746.1          FTTGDKKDFNDFVSRYIKINYELERIVL   267
MEI0609641.1        FTTGDKKDFNDFVSRYIKINYELERIVL   267
MEI0541833.1        FTTGDKKDFNDFVSRYIKINYELERIVL   267
MEI0822658.1        FTTGDKKDFNDFVSRYIKINYELERIVL   267
MEI0619229.1        FTTGDKKDFNDFVSRYIKINYELERIVL   267
MEI0844465.1        FTTGDKKDFNDFVSRYIKINYELERIVL   267
MEI0684780.1        FTTGDKKDFNDFVSRYIKINYELERIVL   267
MEI0820803.1        FTTGDKKDFNDFVSRYIKINYELERIVL   267
SUW05994.1          FTTGDKKDFNDFVSRYIKINYELERIVL   267
WP_013243010.1      FTTGDKKDFNDFVSRYIKINYELERIVL   267
                    ****.** :  :    :.
```

Yellow highlight describes that the amino acid residue found in Chain A and B that interacts with Hit 3 compound is conserved across all the MurI sequences, whereas the green highlight denotes a difference in the amino acid across the genomes (not conserved).
